# Genome-Wide Determination of Gene Essentiality by Transposon Insertion Sequencing in Yeast *Pichia pastoris*

**DOI:** 10.1038/s41598-018-28217-z

**Published:** 2018-07-05

**Authors:** Jinxiang Zhu, Ruiqing Gong, Qiaoyun Zhu, Qiulin He, Ning Xu, Yichun Xu, Menghao Cai, Xiangshan Zhou, Yuanxing Zhang, Mian Zhou

**Affiliations:** 1 State Key Laboratory of Bioreactor Engineering, East China University of Science and Technology, Shanghai, 200237 China; 2 Shanghai Collaborative Innovation Center for Biomanufacturing (SCICB), Shanghai, 200237 China

## Abstract

In many prokaryotes but limited eukaryotic species, the combination of transposon mutagenesis and high-throughput sequencing has greatly accelerated the identification of essential genes. Here we successfully applied this technique to the methylotrophic yeast *Pichia pastoris* and classified its conditionally essential/non-essential gene sets. Firstly, we showed that two DNA transposons, *TcBuster* and *Sleeping beauty*, had high transposition activities in *P*. *pastoris*. By merging their insertion libraries and performing Tn-seq, we identified a total of 202,858 unique insertions under glucose supported growth condition. We then developed a machine learning method to classify the 5,040 annotated genes into putatively essential, putatively non-essential, ambig1 and ambig2 groups, and validated the accuracy of this classification model. Besides, Tn-seq was also performed under methanol supported growth condition and methanol specific essential genes were identified. The comparison of conditionally essential genes between glucose and methanol supported growth conditions helped to reveal potential novel targets involved in methanol metabolism and signaling. Our findings suggest that transposon mutagenesis and Tn-seq could be applied in the methylotrophic yeast *Pichia pastoris* to classify conditionally essential/non-essential gene sets. Our work also shows that determining gene essentiality under different culture conditions could help to screen for novel functional components specifically involved in methanol metabolism.

## Introduction

Large-scale classification between essential and non-essential genes could help to elucidate the molecular underpinnings of many biological processes^1^. Some specific areas such as metabolic research and synthetic biology benefit a lot from essential gene identification in different organisms. Traditionally it was challenging to gather essential gene sets since making knockouts one by one was labor-consuming. Compared with prokaryotic essential genes, the number of eukaryotic essential genes did not exhibit a drastic increase with years, apparently due to the complexity of eukaryotic genome and lack of genome-wide mutagenesis strategies^2,3^.

With the rise of transposon insertion sequencing, essential genes in many pathogenic bacteria have been successfully identified using mariner or Tn5 transposon-mediated mutagenesis system, both of which have high transposition efficiency and low insertional bias^4,5^. This technique was also tried in *Saccharomyces cerevisiae*^6^ and *Schizosaccharomyces pombe*^7^, but has rarely been applied to other eukaryotic organisms to classify conditionally essential/non-essential gene sets.

Transposon insertion sequencing techniques include transposon sequencing (Tn-seq), high-throughput insertion tracking by deep sequencing (HITS), insertion sequencing (INSeq) and transposon-directed insertion site sequencing (TraDIS)^6–11^. These techniques share many similarities and also have some differences^4,5^. For example, Tn-seq and TraDIS were developed when scientists worked on different transposons and several steps such as adaptor designing and ligation were different. Since Tn-Seq has been settled down as the more popular name used by researchers, here we just use this name to describe our transposon insertion sequencing in *Pichia pastoris*.

The methylotrophic yeast *P*. *pastoris* is the most frequently used yeast system for recombinant protein expression^12–15^. Besides, because of its special ability to utilize methanol as sole carbon source, *P*. *pastoris* is also common model to study methanol assimilation and peroxisome synthesis pathways^16,17^. Although the *P*. *pastoris* complete genome has been sequenced in 2009, around one third of the total genes are still annotated as hypothetical with unknown functions^12^. Therefore, studying the conditionally essential gene classification in *P*. *pastoris* under different culture conditions not only helps to improve its gene annotation, but also reveals potential novel targets involved in methanol metabolism and signaling.

Here we tested several transposons and developed a combinational transposon-based mutagenesis system in *P*. *pastoris*. By performing Tn-seq, we analyzed the insertion sites and developed a machine learning method, logistic regression algorithm to predict gene essentiality. We then validated the accuracy of this classification model. Finally we compared the conditionally essential gene sets under glucose and methanol supported growth conditions to screen for novel genes participating in the specific metabolic pathways of methylotrophic yeasts.

## Results

### *TcBuster* and *Sleeping beauty* can transpose in yeast *P. pastoris*

To develop the transposon-based mutagenesis system in *P*. *pastoris*, we tested transposition activities of several transposons including *Himar1* and two Tc1/mariner transposons: *Sleeping beauty* and *Mos1*^18–20^. *Himar1* was one of the most widely used mariner transposon in bacteria, while the other two were used in eukaryotes more frequently. We also checked another two transposons which have been proven to be active in *S*. *cerevisiae*: *Osmar*14 (a rice member of the *Stowaway* superfamily) and *TcBuster* (a flour beetle member of the *Buster* subfamily of *hAT* transposon)^21,22^. All of these transposons target TA sites.

**Figure 1:**
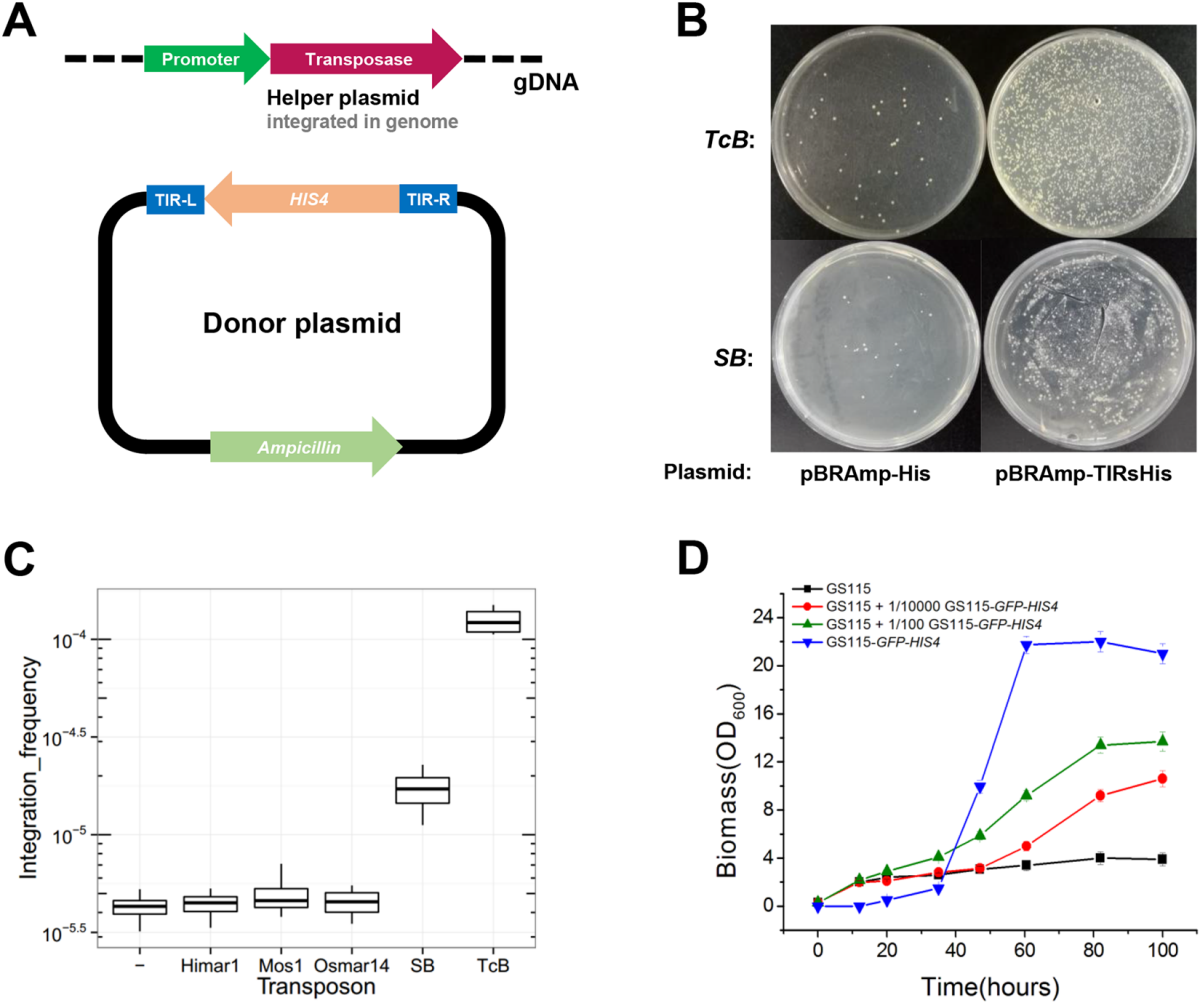
Detection of transposition activities of five common transposons in *P*. *pastoris* and liquid enrichment of transposon mutants. (**A**) A schematic diagram showing the two-component assay system. A helper plasmid integrated into the *P*. *pastoris* genome provides transposase under the control of an inducible promoter. A donor plasmid provides the Transposon Element (TE) lacking transposase but containing a *HIS4*^+^ selection marker. (**B**) YND plates after His^+^ transformant selection. Compared with pBRAmp-TIRsHis, pBRAmp-His lacks the corresponding TIRs which are necessary components for transposition to happen. (**C**) Integration assays of the five common transposons: *Himar1*, *Mos1*, *Osmar14*, *SB* and *TcB* in *P*. *pastoris*. “−” is the negative control which measures the transposition of a *HIS4*^+^ expression cassette from the pBRAmp-His into wild type genome. (**D**) Growth curves of GS115, GS115 + 1/10,000 GS115-*GFP-HIS4*, GS115 + 1/100 GS115-*GFP-HIS4* and GS115-*GFP-HIS4* cells.

We designed a two-component assay system. In this assay, generally a helper plasmid containing the transposase under the control of an inducible promoter was integrated into the *P*. *pastoris* strain GS115. After inducing the expression of transposase for 24 hours, cells were then transformed with a donor plasmid with the histidine auxotrophic marker gene *HIS4* flanked by terminal inverted repeats (TIRs) of indicated transposons (Fig. 1A). Since the donor plasmid lacked *P*. *pastoris* autonomous replication origin, only cells in which transposition event has happened and that have therefore integrated the *HIS4*-containing sequence were able to grow robustly on media without histidine. It is generally known that circular plasmids integrate into the yeast genome at a very low frequency by homologous recombination^23^. As expected, only a small number of colonies grown on YND (histidine^-^) plates two days after the transformation of control donor plasmid lacking TIRs (pBRAmp-His), probably due to the rare integration events of the donor plasmid (Fig. 1B). Oppositely, many more colonies were obtained when pBRAmp-TcBTIRsHis and pBRAmp-SBTIRsHis donor plasmids were used, revealing elegant transposition activities of *TcBuster* (*TcB*) and *Sleeping beauty* (*SB*) (Fig. 1B). We couldn’t see any significant differences of the integration frequency between control and *Himar1*, *Osmar14* or *Mos1* transposons (Fig. 1C), indicating that these transposons were not working in *P*. *pastoris*. To double check whether the colonies were real mutants with transposition events, we randomly picked 20 colonies on pBRAmp-TcBTIRsHis/pBRAmp-SBTIRsHis plates. High-efficiency thermal asymmetric interlaced PCR (hiTAIL-PCR)^24^ was used to amplify the insertion sites together with the unknown sequence nearby. The fact that we observed different patterns of PCR product indicated that *TcB* transposon element (TcBTIRsHis) and *SB* transposon element (SBTIRsHis) were integrated in various genomic loci (Figure S1). (This multiple band phenomenon in each lane is common in hiTAIL-PCR. Since one primer is specific towards transposon element sequence and the other is arbitrary degenerate, it is likely to bind to multiple sites of the genome and generate bands with different lengths. Actually we have tried to pick several bands for sequencing, and it came out with the same chromosome loci.) Sequencing these PCR products confirmed that transposon elements were inserted into different genomic loci (Table S2). We then analyzed the transposition efficiency of *TcB* and *SB*. As shown by Fig. 1C, *TcB* had an integration frequency of ~1.3 × 10^−4^ integrations/cell which was ~30 folds higher than control. *SB* showed a frequency of ~2.1 × 10^−5^ integrations/cell which was ~5 folds higher than control. If we take the electroporation efficiency 1 × 10^−2^ into account, the real integration frequency of *TcB* will be ~1.3 × 10^−2^ integrations/cell, and the real integration frequency of *SB* will be ~2.1 × 10^−3^ integrations/cell.

To obtain a saturated mutant library for Tn-seq, millions of insertion mutants need to be generated. According to the insertion frequency of *SB* (~2.1 × 10^−5^ integrations/cell), around 600 *SB* mutants could be enriched in one 15-cm culture dish, since plating over 3 × 10^7^ cells in one dish is too dense to generate revertant colonies (data not shown). Therefore, thousands of 15-cm culture dishes would be required to generate millions of *SB* mutants, which is quite labor-consuming. To solve this problem, we developed the “liquid enrichment” approach, which could enrich mutants in liquid culture before cells were spread on plates with solid medium. In order to evaluate the growth competition between mutants with transposition events and wild type strain GS115 (*HIS4*^*-*^) in liquid culture without histidine, we transformed GFP-HIS4 construct into GS115 and made the GS115-*GFP-HIS4* strain to mimick the mutants. GS115-*GFP-HIS4* cells carried the GFP reporter which could be separated and counted by flow cytometry. As shown by Fig. 1D and Figure S2, a 10,000 to 1 ratio of GS115 and GS115-*GFP-HIS4* cells (red line, the “GS115 + 1/10,000 GS115-*GFP-HIS4*” group) which best mimicked the cell mixture after transposition induction, has reached twice the biomass of the GS115-only group after 80-hour incubation. Flow cytometric analysis of mixed cells showed that the percentage of GFP positive cells increased to 55.3% after 82 hours and reached 82.1% further after 100 hours. These results indicate that the *HIS4* containing mutants do have growth advantages over GS115 cells when histidine is absent. Therefore, the liquid culture could successfully enrich mutants after 80-100 hours incubation. Finally we were able to collect millions of transposition mutants with only dozens of YND plates.

### Generation and sequencing of mutant libraries by transposon insertion

**Figure 2:**
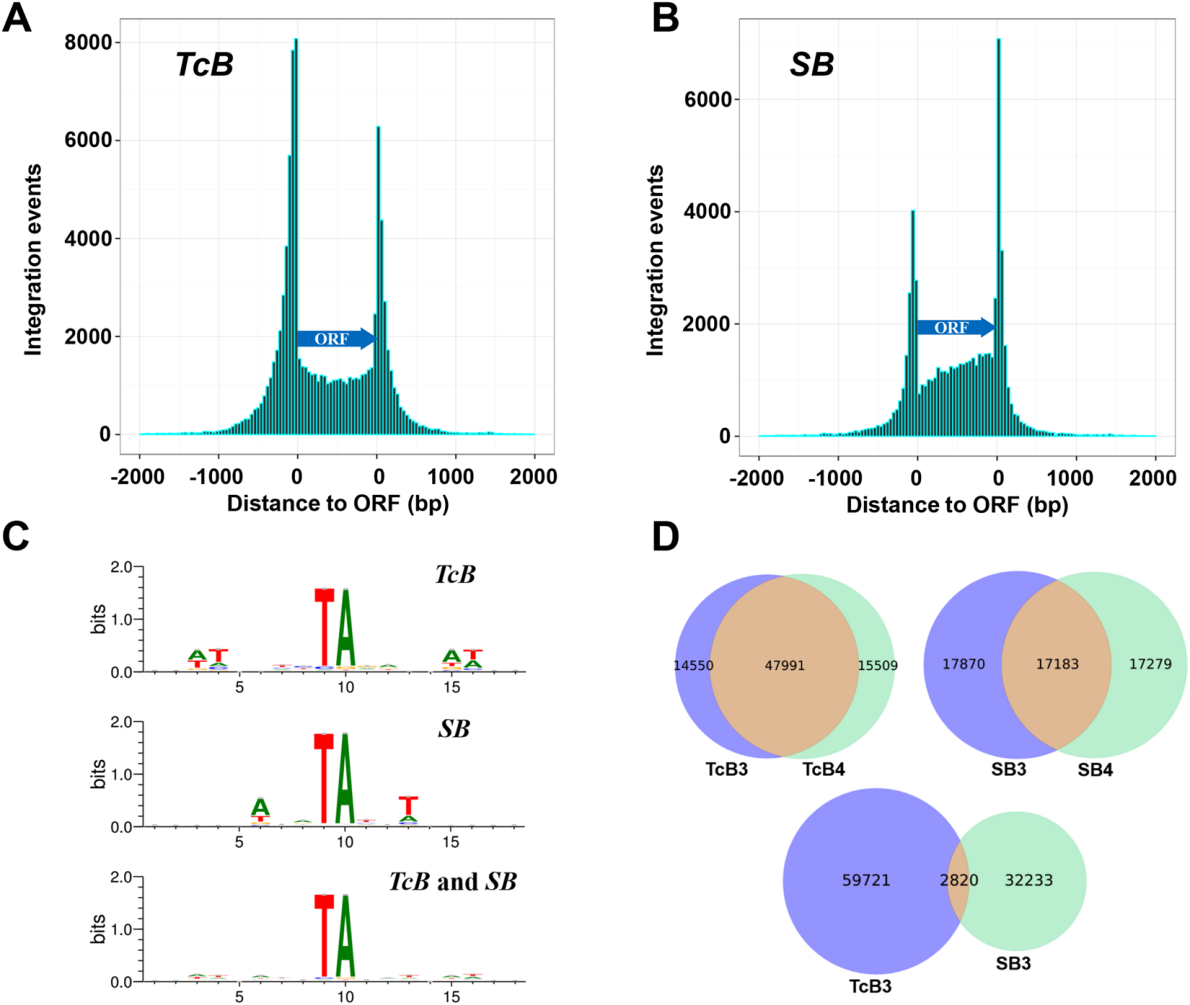
The chromosome target site bias of *SB* and *TcB*. The transposon insertion sites of *TcB* (**A**) and *SB* (**B**) are plotted relative to ORF positions. Each ORF is divided into 25 equal-sized segments and the number of insertions in each segment is displayed. Insertion sites in intergenic regions closer to the 5′ or 3′ end of an ORF are plotted upstream or downstream of the ORFs, respectively. X-axis means distance to ORF while y-axis represents integration events *in vivo*. (**C**) WebLogos generated from insertion sites of *TcB* and *SB* transposons using the website at www.weblogo.berkeley.edu. (**D**) The Venn diagrams showing the number of duplicated insertion sites by two *TcB* libraries, two *SB* libraries, as well as one *TcB* library and one *SB* library.

Using the two-component assay system and “liquid enrichment” approach, we generated ten independent *TcB* insertion pools and ten independent *SB* insertion pools, with an average of ~300,000 and ~150,000 insertional mutants per pool, respectively (Table S3). Following genomic DNA (gDNA) isolation, the transposon-gDNA junctions from each pool were subsequently captured, subjected to PCR amplification, and then sequenced by Illumina HiSeq X Ten (*Materials and Methods*). According to the genomic sequences adjacent to the TIRs, the insertion sites in mutant libraries could be mapped. As a result, 10 *TcB* insertion sets (Insertion pool *TcB*1-10) and 10 *SB* insertion sets (Insertion pool *SB*1-10) were collected. We merged 10 *TcB* sets into a *TcBall* set containing 133,061 unique insertion sites, and merged 10 *SB* sets to produce a *SBall* set containing 79,296 unique insertion sites. Then we investigated the distribution of these insertions in exons, introns and intergenic regions (IGRs). Although only 19.8% of the *P*. *pastoris* genome was defined as IGRs, over 40% insertions were distributed in IGRs (48.06% for *TcB* and 41.41% for *SB*) (Figure S3A). Since few genes contained introns in *P*. *pastoris* and insertions were rarely distributed in introns (0.66% for *TcB* and 0.46% for *SB*), we then grouped insertions in both exons and introns as insertions in ORFs. To survey the positional bias of transposon insertions, we plotted the distribution density of insertions in IGRs and ORFs (Fig. 2A,B). Although there was a slight difference between two transposons, peaks of insertions in IGRs close to the 5′ or 3′ end of ORFs were observed in both. This type of insertional bias also occurred in *S*. *cerevisiae* and *S*. *pombe*, which was believed to be caused by the nucleosome occupancy in yeast genome^7,25^.

Except for the strong preferences for TA, *TcB* and *SB* transposons may still have different biases on the nucleotide sequences containing TA sites. To investigate into this, we analyzed the sequences at insertional junctions to reveal any peripheral preference besides the TA target site. Figure 2C shows the web logos generated for *TcB* and *SB* recognition sites, and the proportions of the four nucleotides at particular sites are presented in Table S4. For *TcB*, there were some visible preferences such as T at −5, +6 positions and A at −6, +5 positions, which was similar to what Li *et al*. observed in HeLa cells^22^. However, *SB* exhibited totally different preferences which was A at −3 position and T at +3 position. The web logos generated by combined *TcB* and *SB* data weakened the insertional bias (Fig. 2C, bottom panel). This informs us that combining the two transposons could further decrease insertional bias and increase the mutant library complexity. The Venn diagrams generated by one *TcB* subset and one *SB* subset also support this (Fig. 2D). Libraries generated by the same transposon shared lots of same insertion sites, whereas this number decreased a lot when different transposons were used, mainly due to the different insertional preference.

To investigate the saturation of the library, we merged one *TcB* set and one *SB* set into a new dataset called *T&S*, so that we had 10 *T&S* datasets each with approximately 100,000 mutants. We determined the cumulative density of insertions with the 10 library sets (in random order) and found that the increasing slope became quite flat after more than 8 library sets were pooled together (Figure S3B), suggesting that our 10 library sets were near saturation. The *TSall* dataset generated by merging 10 *T&S* sets contained 202,858 unique insertions (Dataset S1), with an average density of 22 insertions per kilobase.

### Identification of conditionally essential genes in *P*. *pastoris* under glucose supported growth condition

**Figure 3:**
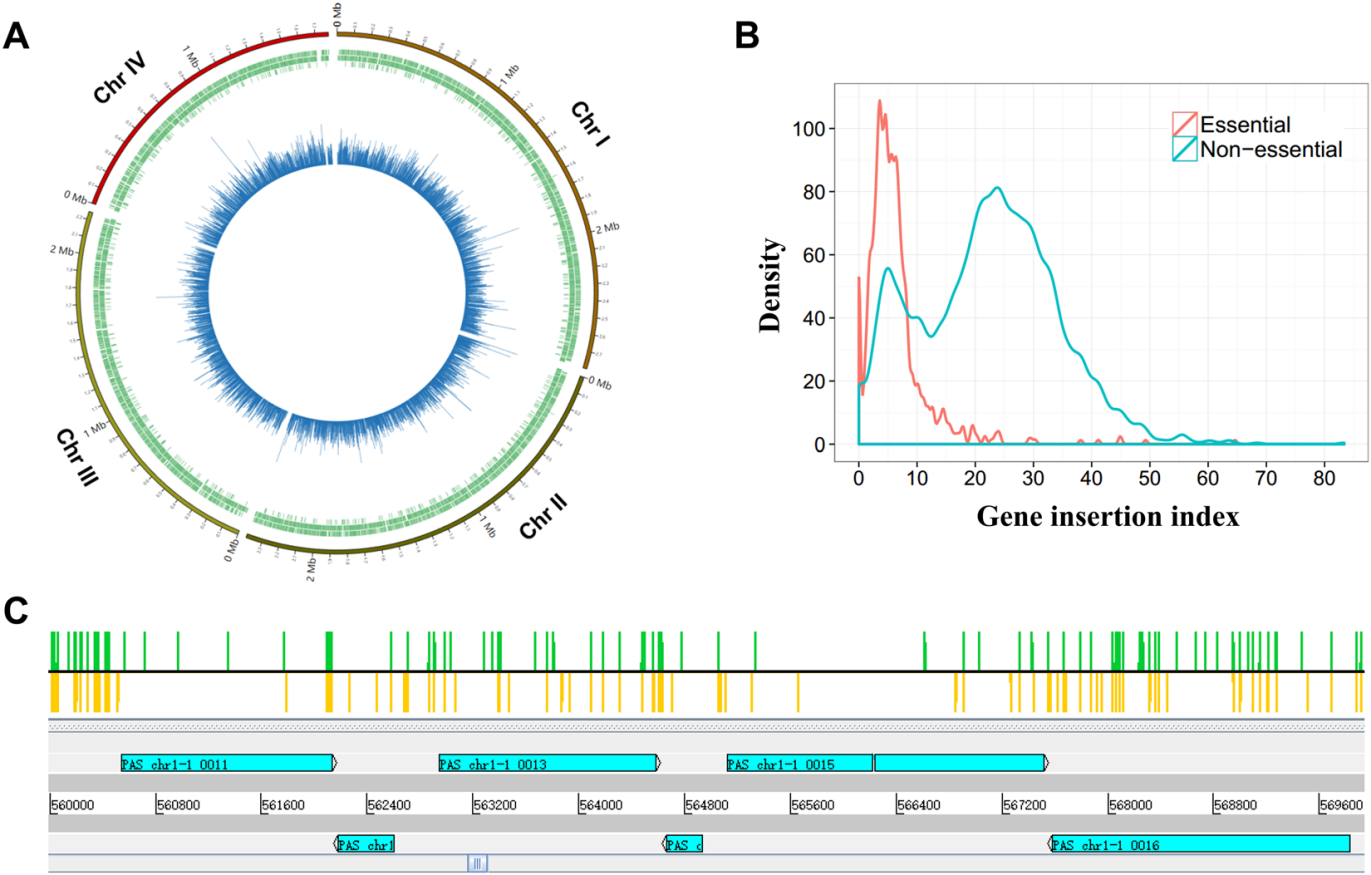
The distributions of transposon insertions in each annotated gene of *P*. *pastoris*. (**A**) Distribution of the ORF and transposon insertions on *P*. *pastoris* genome map. The green bars indicate ORFs of *P*. *pastoris* GS115 strain, and the blue bars indicate the gene insertion index (GII) values of each ORF. (**B**) The distributions of GII are plotted for genes whose orthologs are known to be essential (red) or non-essential (blue) in *Saccharomyces cerevisiae* and *Schizosaccharomyces pombe*. The y-axis “Density” here means the GII distribution density, and the area under the curve represents gene number under a certain GII range. The curves were plotted based on 753 possibly essential genes and 2180 possibly non-essential genes mentioned in the main text. (**C**) Transposon insertion sites from a small section of the *P*. *pastoris* GS115 chromosome I, showing that the possibly essential genes (*PAS_chr1-1_0011* and *PAS_chr1-1_0015*) sustain significantly less transposon insertions than the possibly non-essential genes (such as *PAS_chr1-1_0013* and *PAS_chr1-1_0016*). The green bars indicate forward insertions and the yellow bars indicate reverse insertions.

The *TSall* dataset harboring high-density insertions affords the opportunity to identify potential and conditionally essential genes since essential genes are expected to have fewer insertions than non-essential genes^26,27^. Therefore, to determine gene essentiality, we mapped the insertions in *TSall* library to each annotated gene. Certainly, the number of insertions per gene is affected by gene length or the number of TA sites in a gene^28,29^. Since the overall correlation between TA sites and insertions per gene was 0.68 (*R*^2^), which was slightly better than gene length versus insertions per gene (*R*^2^ = 0.64) (Figure S4), we used TA sites as a normalization factor and created a Gene Insertion Index (GII) for every annotated ORF by normalizing the insertions in each gene. We plotted the GII and position of each ORF onto the four chromosomes to look at the insertion profile of each gene. As shown by Fig. 3A, different ORFs possessed distinct GII (Detailed GII values for each gene are listed in Dataset S4). However, at the genomic level, GII was quite evenly distributed across four chromosomes, indicating that there was no replication origin-proximal insertion bias which was found prevalent in bacteria^5,30^.

To investigate whether the GII could be suitable to distinguish essential and non-essential genes, we analyzed the GII of 753 possibly essential genes which were orthologs of *S*. *cerevisiae* and *S*. *pombe* essential genes, and the GII of 2180 possibly non-essential genes which were orthologs of *S*. *cerevisiae* and *S*. *pombe* non-essential genes (Dataset S2). Both essential and non-essential genes in *S*. *cerevisiae* and *S*. *pombe* were from the highly verified database (DEG) (Constructed through the gene knockout method, http://www.essentialgene.org/)^2,31,32^. Apparently, the GII values of most possibly essential genes were lower than most possibly non-essential genes, with a peak at 3.5 (Fig. 3B, red and blue curves). Density distribution of GII values from possibly non-essential ORFs appeared in a bimodal curve in which the leftmost sharp peak probably indicated some “fake” non-essential genes which were required for cell growth under YND medium. And the rightmost peak likely represented real non-essential genes which could be wilfully mutated without affecting the viability of the cell. More concretely, we examined the distribution of insertions in ORFs further and found that the possibly non-essential genes (such as *PAS_chr1-1_0013* and *PAS_chr1-1_0016*) always had an even distribution of insertions across the whole gene, whereas the insertions of possibly essential genes (such as *PAS_chr1-1_0011* and *PAS_chr1-1_0015*) were mainly located at the edge areas, especially in the terminal region which was believed not to influence gene function (Fig. 3C). However, a few possibly essential genes still had insertions in their middle regions which were usually parts of core functional domains, probably due to insertions in one copy of a transiently duplicated gene^33^. It’s also possible that only a small fraction of the CDS is actually essential in these genes^6^. For example, *PAS_chr1-1_0015*, *PAS_chr1-1_0035* and *PAS_chr1-3_0239* have insertions except some key domains (Figure S5).

Since many existing algorithm models determining gene essentiality are from bacteria and are developed based upon the principle “essential gene regions will not contain transposon insertions”^5,33,34^, building a novel predictive model which is able to effectively assess gene essentiality in yeast becomes necessary. Recently, machine learning has been applied in a broad range of areas within genetics and genomics^35^. Machine learning contains lots of algorithms for classification, such as logistic regression, support vector machines and K-nearest neighbors. In this study, using GII as a main factor, we developed a supervised learning method logistic regression algorithm to classify essential and non-essential genes. Supervised learning was based on the idea that an algorithm trained labelled datasets could be used to predict unlabelled datasets *(Materials and Methods*). For training, we used 161 essential genes which were orthologs of *S*. *cerevisiae* and *S*. *pombe* essential genes^2,31,32^ with the lowest E-value, and 171 *P*. *pastoris* non-essential genes which were successfully knocked out previously (Dataset S3) as input datasets. The Expect value (E) is a parameter that describes the number of hits one can “expect” to see by chance when searching a database of a particular size (https://blast.ncbi.nlm.nih.gov/Blast.cgi?CMD=Web&PAGE_TYPE=BlastDocs&DOC_TYPE=FAQ#expect). E-values here were calculated by the BLAST program from NCBI. The GII values and reads densities of the 332 (161 + 171) labelled samples were assigned to the feature matrix X and the corresponding class labels of the gene essentiality to the vector y. Then we randomly split the X and y arrays into 70 percent training data (232 samples) and 30 percent testing data (100 samples). Having trained the logistic regression model for 10 times, we could see that our model exhibited a relative stable prediction accuracy of ~96%. It is likely that the misclassification on the testing dataset was partially caused by the bias or saturation of our transposon system, as well as the wrong labeling of some samples in the training set. To investigate the effect of mutant library saturation on classification accuracy, we randomly extracted 7 subsets from *TSall* dataset with different sizes and subsequently trained these 7 subsets with the machine learning approach. As expected, the accuracy score increases with library subset numbers at the beginning, and reaches a plateau after mutant library exceeds a density of 170,000 independent insertions (Figure S6). This indicates that our *TSall* library is large enough to get a stable and high classification accuracy.

Based on the machine learning training result of *TSall* dataset, we developed a mathematical algorithm which could calculate the probability value of non-gene essentiality (NEP) (see *Materials and Methods*, the closer the NEP is to 1, the more likely the gene is to be a non-essential gene; the closer the NEP is to 0, the more likely the gene is to be an essential gene). Subsequently, we determined gene essentiality for 5,040 annotated genes and the results were listed in Dataset S4. To increase model reliability, we excluded 195 genes with fewer than fifteen TA sites, as well as the *HIS4* gene which was used as transposon maker. According to the criteria of our logistic regression algorithm, of the remaining 4,844 genes, 1,968 with NEP less than 0.5 were deemed likely essential. However, 15 of 887 genes with a NEP of 0.03–0.5 were known non-essential genes, indicating that the NEP of genes in the range 0.03–0.5 were ambiguous. In contrast, 2,876 genes with NEP more than 0.5 were deemed likely non-essential, and the NEP of genes in the range 0.5–0.9 were also seemingly ambiguous because 5.0% of 478 genes with NEP in this range were orthologs of *S*. *cerevisiae* and *S*. *pombe* essential genes. To reach an essentiality assignment with higher confidence, 1,054 genes with NEP less than 0.03 were predicted to be putatively essential, whereas 2,393 genes with NEP > 0.9 were categorized as putatively non-essential genes. Genes with NEP between 0.03 and 0.5 were put in the ambig1 category which presumably comprised many genes essential for YND condition and some genes advantageous for growth; genes with NEP between 0.5–0.9 were put in the ambig2 category. This category presumably comprised genes advantageous for growth but non-essential as well as small genes whose essentiality could not be determined confidently by our method. Ambig1 and ambig2 categories could also comprise essential genes that contain non-essential domain. All of the classified essential/non-essential genes above were actually conditionally essential/non-essential, since Tn-seq was performed under YND condition.

### Validation of the classification model

In order to validate our classification model for predicting conditionally essential genes, we employed bioinformatic strategy (homologous alignment with other two model yeasts) together with experimental strategies to evaluate its accuracy.

### Comparing our classification with the reported essential gene sets in *S*. *cerevisiae* and *S*. *pombe*

**Figure 4:**
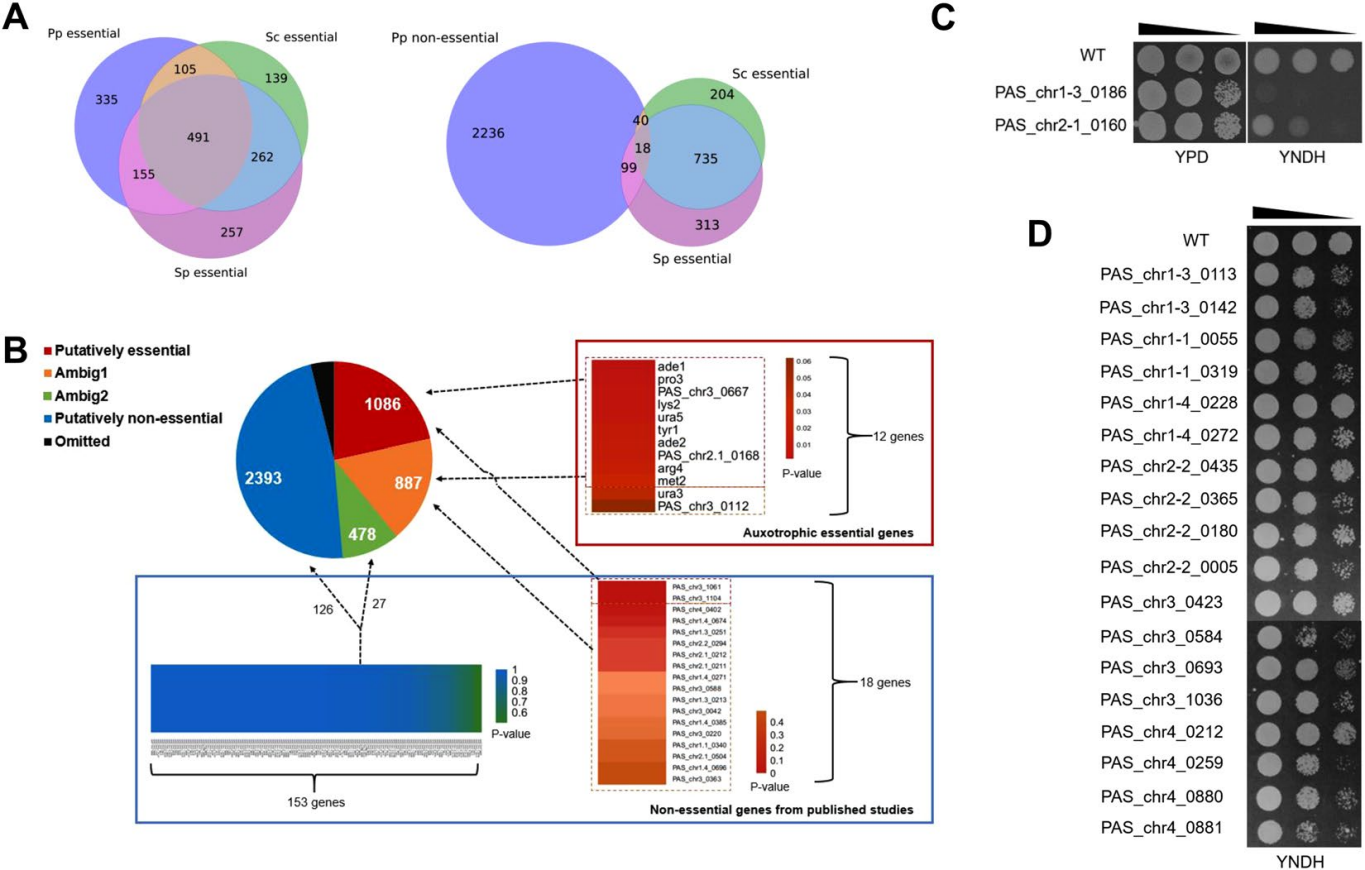
Validation of our classification model. (**A**) Left panel: a Venn diagram showing the overlap among putatively essential genes predicted in *P*. *pastoris* (YND condition), reported essential genes in *Saccharomyces cerevisiae* and in *Schizosaccharomyces pombe*. Right panel: a Venn diagram showing the overlap among putatively non-essential genes predicted in *P*. *pastoris* (YND condition), reported essential genes in *Saccharomyces cerevisiae* and in *Schizosaccharomyces pombe*. (**B**) Essentiality prediction by our model of the 171 non-essential genes (blue frame) and 12 essential genes (red frame) identified previously. (**C**) The growth rates (shown by spotting assay) of *PAS_chr1-3_0186* and *PAS_chr2-1_0160* mutants on YPD and YNDH solid medium. (**D**) The growth rates (shown by spotting assay) of 18 putatively non-essential genes on YNDH solid medium.

We compared our classification in *P*. *pastoris* to the highly verified database of essential genes (DEG) of *S*. *cerevisiae* and *S*. *pombe*^2,31,32^. As shown by Fig. 4A (left panel), 491 putatively essential genes were orthologs of essential genes in both yeasts. This overlapped group represented a highly credible subset of essential genes, and we referred them as “general” essential genes in yeast. Another 260 genes also got a certain degree of verification since their orthologs were either essential in *S*. *cerevisiae* or in *S*. *pombe*. An additional subset (skeptically essential subset) consisting of 335 genes outside two yeast sets was presumably comprised by *P*. *pastoris*-specific essentials, unrecognized homologs, auxotrophic gene and any false assignments that may have been included. For the putatively non-essential group, almost all (93.4%) genes were outside the essential genes sets from *S*. *cerevisiae* or *S*. *pombe* (Fig. 4A, right panel), with only 18 genes located in the overlapped area.

### Model validation with previously reported essential and non-essential genes

To validate our classification model, we also combined experimental evidence for the gene essentiality in YND. As shown by Fig. 4B, of the 171 non-essential genes from published studies (They are believed to be non-essential since the knockouts could grow on YND), 126 were successfully classified as non-essential genes by our model. Of the 45 remaining genes, 27 were classified as ambig2, 16 were classified as ambig1 and 2 were classified as essential genes (*PAS_chr3_1061* and *PAS_chr3_1104*). As reported by Shen *et al*.^36^, many genes classified into ambiguous categories were advantageous for growth, such as *PAS_chr1-4_0271*, *PAS_chr1-3_0213* and *PAS_chr3_0042*. Besides, we double checked *PAS_chr3_1061* and *PAS_chr3_1104* mutants, and found that only the latter one is a real knockout. Therefore, the significant error rate of our model is 1 in 171. To examine the classification of essential genes, we checked 12 genes whose mutation resulted in auxotrophy (They are believed to be essential since the knockouts could not grow on YND)^36,37^. As a result, 10 out of 12 were successfully predicted as essential genes and 2 were classified as ambig1.

### Model validation by constructing gene knockouts using the CRISPR/Cas9 system

To conclusively determine whether the predicted genes were required for cell growth in YND, we constructed frameshift mutant strains based on CRISPR/Cas9 gene-targeting technology. A CRISPR/Cas9 construct, which bearing RNA Pol II promoters, ribozymes and a human codon optimized Cas9, was applied to disrupt the indicated genes (*Materials and Methods*). For most loci, the observed targeting efficiencies are close to 100% in *P*. *pastoris*^38^. Taking into account that some conditionally essential genes may be auxotrophic, CRISPR/Cas9 gene-targeting was conducted in YPD rich medium and cell growth was further tested on YNDH.

First of all, we picked the 18 genes from the putatively non-essential gene group and which were also orthologs of both *S*. *cerevisiae* or *S*. *pombe* essential genes (Fig. 4A, right panel, central area). We tested these genes since they had a larger chance to be mistakenly predicted as non-essential genes in *P*. *pastoris*. These genes were all successfully disrupted by the effective targeting of CRISPR/Cas9 system (Table S5**)**, and the corresponding mutant strains were able to grow in YNDH (Fig. 4D). Therefore, their identity as non-essential genes was validated.

Next, we chose 20 putatively essential genes from the “skeptically essential subset” with relatively high NEPs and tried to validate them one by one. Among the 20 genes, *PAS_chr1-1_0389* had already been reported to be the arginine auxotrophic gene^37^ and its mutant failed to grow in YNDH. Therefore, we continued to test the rest 19 genes. Compared with non-essential genes, validating essential genes was a little bit tricky, since the rationale was based on the failure to obtain grown mutants. For each putatively essential gene to be verified, we sequenced at least 30 colonies to ensure three or more effective targeting (The average effective targeting ratio of the CRISPR/Cas9 system here was larger than 10%). As a result, we were only able to obtain frameshift mutants of *PAS_chr2-1_0160* and *PAS_chr1-3_0186* in YPD (Table S5). The *PAS_chr1-3_0186* mutant failed to grow on YNDH, suggesting that *PAS_chr1-3_0186* was an auxotrophic gene (Fig. 4C). The *PAS_chr2-1_0160* mutant only showed affected growth on YNDH, indicating that our prediction still had a certain error rate.

Again, the predicted essential/non-essential genes mentioned above were also conditionally essential/non-essential in the tested medium.

### Identification of conditionally essential genes under methanol supported growth condition and screening for novel methylotrophic functional genes

**Figure 5:**
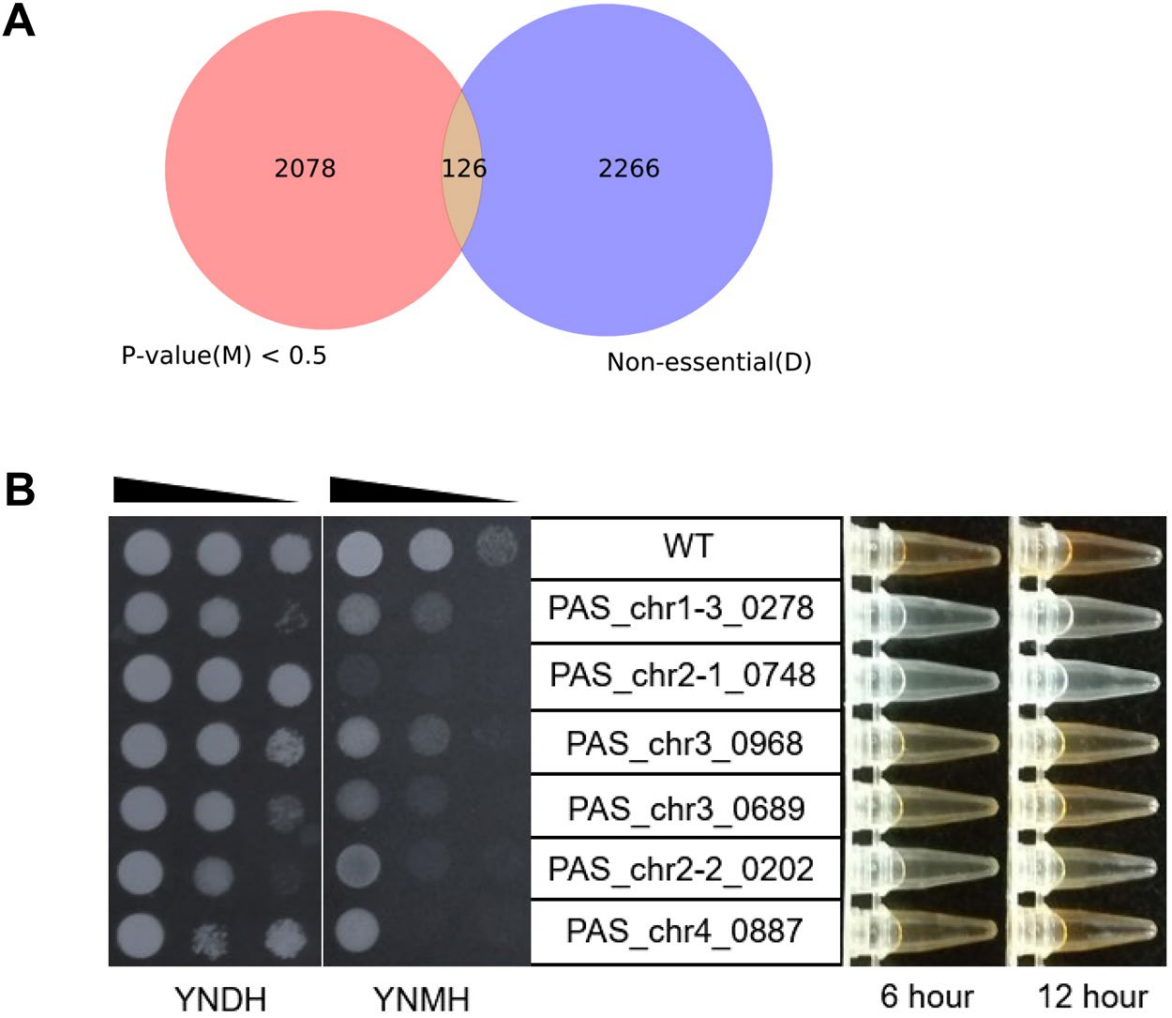
Identify essential genes specifically involved in methanol supported cell growth. (**A**) A Venn diagram showing the overlap between putatively essential genes with NEP < 0.5 in methanol condition and putative non-essential genes in glucose condition (NEP > 0.5). (**B**) The growth rates (shown by spotting assay) and Aox enzymatic activities (shown by the colorimetrical assay) of the six mutants in glucose and methanol cultured conditions.

To identify genes required for optimal growth of *P*. *pastoris* under methanol, aliquots of transposon insertion pools generated from YND medium were re-inoculated into fresh YNM medium at OD_600_ of 0.05 and grown at 30 °C with shaking at 200 rpm to an OD_600_ of ∼6 (approximately seven generations). In order to remove the insertion mutants which could grow at glucose condition but not methanol thoroughly, we repeated re-inoculation twice. Subsequently, the insertion pools from YNM (Named *TSall-M*) were high-throughput sequenced with the Tn-seq approach and ~120,000 unique insertion sites were detected. Again we calculated the NEP of gene essentiality using the logistic equation which was generated by machine learning training before (*Materials and Methods*). The results are shown in Dataset S5. Using Tn-seq data to excavate condition-specific essential genes is often accomplished by directly comparing insertion status in the initial library and after growth in a specific condition^5,39^. To screen for genes required for methanol metabolism but not for glucose utilization, we picked the putatively non-essential gene group under YND (*P* > 0.9) and tried to detect any gene required for advantageous growth in YNM (*P* < 0.5 was set here) (Fig. 5A). As a result, 126 genes from the overlapped area are potentially specific genes required for methanol metabolism (Dataset S5).

As expected, these 126 genes included some previously reported genes involved in methanol utilization pathway (*PAS_chr3_0841*/*DAK*), peroxisome assembly (*PAS_chr2-2_0186*/*PEX5*, *PAS_chr3_1045*/*PAS1* and *PAS_chr4_0496*/*PAR4*), and transcriptional regulation of *AOX1* promoter (*PAS_chr4_0203*/*TRM1* and *PAS_chr3_0836*/*MIT1*)^12,16,40–43^. However, many of the rest genes lacked experimental evidences of their participation in methanol metabolism. Since we were particularly interested in genes involved in transcriptional regulation of *AOX1* promoter, kinase genes involved in methanol signaling, and hypothetical genes with no orthologs in *S*. *cerevisiae*, we chose four putative transcription factors, two kinases and two hypothetical proteins and tested their essentiality under methanol (Table S6). Among these 8 genes, the mutants of two kinase genes *PAS_chr3_0072* and *PAS_chr1-4_0498* showed severe growth defects in the presence of methanol, as revealed by our previous study^36^. For the rest 6 genes, their mutants all showed certain extent of growth defects under methanol (Fig. 5B), especially the *PAS_chr2-1_0748* mutant. Annotated as hypothetical protein with a Glutathione S-transferase domain, mutant with this gene could not grow on methanol plate and showed no alcohol oxidase (Aox) enzymatic activity. Besides, the *PAS_chr1-3_0278* and *PAS_chr2-2_0202* mutants also had severe growth defects and low Aox enzymatic activities on methanol. These genes may play a more important role in methanol-specific signaling or utilization.

## Discussion

Transposon insertion sequencing technique has led to significant advancements on essential gene research and functional gene screening. As for essential gene identification, so far most studies were done in bacteria with the mature mariner derived or Tn5 derived transposon mediated random insertion system. Transposon based random insertion and subsequent Tn-seq has rarely been tried in eukaryotic systems to classify essential genes^6,7^. Here, we tested five different transposons in yeast *P*. *pastoris* and successfully detected transposition events after the *TcB* or *SB* transposon system was introduced, with a net integration frequency ~1.3 × 10^−2^ and ~2.1 × 10^−3^, respectively. We also developed a “liquid enrichment” approach to increase the density of transposon mutants and save labor. By merging the *TcB* and *SB* insertion libraries and performing Tn-seq, we identified a total of 202,858 unique insertions under glucose supported growth condition. We then developed a machine learning method, logistic regression algorithm to classify the 5,040 annotated genes into putatively essential, putatively non-essential, ambig1 and ambig2 groups. Existing data and additional mutants were created to validate this classification model. Besides, Tn-seq was also performed under methanol supported growth condition and methanol specific essential genes were identified. By comparing with non-essential genes under glucose condition, we were able to locate several interesting targets which were potentially novel components involved in methanol metabolism and signaling. Future work on these targets may help to reveal important regulatory mechanisms under methanol.

Our findings suggest that transposon mutagenesis and Tn-seq could be applied in the methylotrophic yeast *P*. *pastoris* to classify conditionally essential/non-essential gene sets. This technique is also likely to work in other methylotrophic yeasts or other eukaryotes. By comparing the conditionally essential gene sets in methylotrophic and non-methylotrophic yeasts, it is possible to reveal the role of evolution in designing methanol metabolism and signaling. Besides, usually genome-wide knockout and engineering are rarely done in “non-conventional” organisms because of limitations in funding resources and labor. Here we find that the transposon mutagenesis and Tn-seq is simple to be applied in a new organism and helped to classify conditionally essential genes with much less labor consumption. Besides methanol, transposon insertion sequencing may also be a good tool to examine conditionally essential genes under other culture conditions.

The number of total TA sites in *P*. *pastoris* genome is 544674, and the number of TA sites we targeted is 202858. Therefore, our targeting percentage is around 40%. This percentage was around 30% (360513/1302408) in *S*. *pombe*^7^. There are two reasons explaining why this percentage is not very high in eukaryotes: 1. Although *TcB* and *SB* transposons recognize TA sites, they still have some preferences on the sequences around TA (Figure 2C); 2. Many TA sites may not be easy to approach in eukaryotes, with the binding of histones or other proteins.

The insertions from *TcB* and *SB* transposons were more enriched in intergenic regions (Fig. 2A). This type of insertional bias was presumably consistent with nucleosome-free regions instead of target site choice by the transposase itself, which was consistent with the insertional distributions of the *Hermes* transposon in *S*. *cerevisiae* and *S*. *pombe*^7,25^, and *piggyBac* transposon in human CD4^+^ T cells^44^.

Previously gene length was a widely used normalization factor when calculating insertion frequency. However, here we used TA sites instead of gene length to normalize Tn-seq data and created GII, since the overall correlation between TA number and insertions (R^2^ = 0.68) was better than gene length vs insertions (R^2^ = 0.64).

In principle, the starting transposon pool will not contain disruptions in any of the genomic regions that are required for growth^5^. However, here we could still find a few insertions at the 5′ end and middle regions of some essential genes, indicating that some essential loci could sustain different extents of insertions. Besides, we also found that the sequencing reads number of each insertion site had a certain level of disorder, which could not reflect the fitness of the loci accurately. Therefore, developing a machine learning algorithm will be a good choice to analyze this type of black box model. As a widely used expression system, *P*. *pastoris* has a few reported essential and non-essential gene resources, which created the necessary foundation for training and testing. We then combine logistic regression to predict the probability of a particular event.

Through validation procedures, we find that our model designed for gene essentiality prediction is effective, but still has a low false positive rate. For example, of the 171 non-essential genes from published studies, one gene (*PAS_chr3_1061*) was misclassified into the essential category (Fig. 4B); and of the 20 putatively essential genes from the “skeptically essential subset” with relatively high NEPs, we also found one gene (*PAS_chr1-3_0186*) was not required for growth on YND (Fig. 4C). The reason to have a false positive rate is likely because our model was developed based on statistical analysis training, as well as some inevitable limitations of Tn-seq^5,45,46^. Even genome-wide deletion assay will still have a false positive rate^7,47^, and the rate is acceptable as long as it is smaller than 0.05.

Our putatively essential gene set was screened in YND medium rather than YPD rich media. Thus, this list must include auxotrophic genes whose mutants could not grow in YND because of lacking amino acids, nucleotides or other nutrients. It will be simple to locate these genes by comparing Tn-seq profiles in YPD and YND.

4133 *P*. *pastoris* genes have orthologs in *S*. *cerevisiae*. Therefore, we compared gene essentiality predictions in these *P*. *pastoris* genes with their *S*. *cerevisiae* orthologs^2,6,32^ (Dataset S6). For those *P*. *pastoris* genes divided into essential groups, 60% of them have the same classification with their *S*. *cerevisiae* orthologs; for *P*. *pastoris* genes divided into non-essential groups, 97% of them have the same classification with their *S*. *cerevisiae* orthologs. The discrepancies are likely to be caused by different culture conditions and different ways to analyze data. More importantly, distinct features of *P*. *pastoris* and *S*. *cerevisiae* may offer different functions and essentiality on orthologous genes. As for those *P*. *pastoris* genes classified into ambiguous groups, referring to the classifications of their *S*. *cerevisiae* orthologs may be a good way to provide more information for further study. Besides, included in our list there are 491 genes whose orthologs are also essential in both *S*. *cerevisiae* and in *S*. *pombe* (Fig. 4A, left panel, central region; Dataset S6). These “general” essential genes may reflect the most basic processes required for yeasts’ viability and growth, such as gene transcription, proteins translation and primary metabolism. These highly conserved genes will give us more ideas on the importance of their orthologs in higher eukaryotes including mammals. In contrast to “general” essential genes, orthologs of some essential genes in *P*. *pastoris* are not essential in *S*. *cerevisiae* and in *S*. *pombe*. These genes are likely specific *P*. *pastoris* essential genes and may play a necessary role in methylotrophic yeast. Anyway, our classification of essential and non-essential genes provides information on every single gene in the *P*. *pastoris* genome by predicting whether deleting a gene of interest would be viable or not. We generate more useful resources for the *P*. *pastoris* research community. Since we located several potential targets involved in methanol signaling and metabolism, further studies are needed to study their detailed functions.

## Materials and Methods

### Strains and culture condition

*P*. *pastoris* GS115 (Invitrogen), a histidine-deficient strain, was used for transposition activity assay, construction of the Tn-seq library and CRISPR/Cas9 editing assay. Medium for *P*. *pastoris* culture used in all experiments contained abundant nitrogen source, which was beneficial to the stability of haploid state. These medium included: YPD (1% yeast extract, 2% peptone and 2% glucose); YND (0.67% yeast nitrogen base without amino acids [YNB] and 1% glucose); YNDZ^r^ (YND supplemented with bleomycin zeocin at 100 μg/ml); YNDH (YND supplemented with histidine at 50 μg/ml); EMM (Edinburgh minimal medium without Vitamin B1, and 1% glucose); YNM (0.67% YNB and 0.5% methanol). For plates with solid medium, 2% agar powder was added. The *Escherichia coli* strain TOP10 used for plasmid propagation was cultivated at 37 °C in LB medium (1% tryptone, 0.5% yeast extract, and 0.5% NaCl). When necessary, ampicillin, or zeocin was added into LB medium at a final concentration of 100 or 50 μg/ml, respectively. All yeast strains were cultured at 30 °C while *E*. *coli* strains were cultured at 37 °C.

### Plasmid construction

All plasmids for transposition assay generated in this study were constructed using DNA recombination method following the instruction of NEBuilder® HiFi DNA Assembly Cloning Kit (NEB #E5520S). Primers, templates and PCR amplification products for plasmid construction are listed in Table S1. In brief, the helper plasmids were constructed by assembling promoter fragment, transposase coding DNA fragment, and AOX1TT-Zeocin-pBR322 fragment; the donor plasmids were generated by assembling pBR322-Amp fragment, transposon left-TIR (TIRL) fragment, *HIS4* expression cassette and transposon right-TIR (TIRR) fragment. Promoter fragment refers to *MET3p*, *THI11p* or *AOX1p* promoter fragment. Transposase coding DNA fragment, TIRL and TIRR fragment are from corresponding transposons including *Himar1*, *Sleeping beauty*, *Osmar*14, *TcBuster* and *Mos1*, respectively.

For construction of plasmids used in CRISPR/Cas9 editing assay, we cloned HsCas9 and gRNA from pPpT4_pHTX1-HsCas9-GUT1-gRNA2 vector^38^. Briefly, pAG32Linear and HXT1-HsCas9 fragments were used to generate pGA32-HsCas9 plasmid by DNA recombination; pDZ-Zeocin fragment, PARS fragment and pDZ-Zeocin fragment were used to generate pBRPARS2Zeo-gRNA-GUT1 plasmids (Details are shown in Table S1).

### The two-component assay

To construct the two-component assay system, the helper plasmid was linearized by either restriction endonuclease or PCR amplification of the entire construct (with PrimeSTAR^®^ Max DNA Polymerase R045A Takara) when suitable cleavage site was not available. Then the linearized plasmid (2 μg) was transformed by electroporation into haploid wild-type strain GS115 (OD_600_ = 100, 100 μL). Zeocin resistance transformants were isolated on YPDZ^r^ plates. The positive transformants with integration at the correct locus were confirmed by PCR analysis and further Sanger sequencing. The cells harboring the transposase gene regulated by different promoters (P_*MET3*_, P_*THI11*_ and P_*AOX1*_) were incubated with YND, EMM or YNM medium for 24 hours, respectively. Then the donor plasmid (3-5 μg) were transformed into these cells (OD_600_ = 100, 100 μL) to create transposition events. After electroporation, 1 mL YND and 1 mL sorbitol were added and cells were incubated for 2 hours for recovery. Then 500 μL cell culture was spread on a 15-cm YND plate lacking histidine and incubated for 48 hours.

*For *P*. *pastoris* cells, OD_600_ = 1 usually suggests a concentration of 1 × 10^7^ cells per mL (experimentally measured).

### CRISPR/Cas9 editing assay

The linearized pGA32-HCas9 plasmid was integrated into GS115 genome first, and then circular plasmid pBRPARSZeo-gRNA-(target sequence) was transformed into cells after inducing for 24 h. It is worth noting that, the insertion of “target sequence” was performed by changing 20 bp of the GUT1 gRNA using a PCR-mediated method of extending overlapping gene segments. The “target sequence” we used here and the corresponding primers used to clone “target sequence” are listed in Table S1. Subsequently, the Zeocin resistant transformants were picked into YPDZ^r^ liquid medium to and cultured until the cell concentration reached OD_600_ of 5–12. Then we used the corresponding primers (Table S1) designed for validation of CRISPR/Cas9 targeting to PCR amplification, followed by Sanger sequencing.

### Identification of transposon insertion sites using hiTAIL-PCR

To identify the insertion sites of *TcB* and *SB*, two sets of each two primers specific for the *TcB* and *SB* right-TIR regions (TcB-SP1 [5′-AAAGCACGGGCTCACCTTTTCG T-3′], TcB-SP2 [5′-TGTCCCTAAAATCTCATCTGGGTGT-3′], and SB-SP1 [5′-GA GTGTATGTAAACTTCTGACCCAC-3′], SB-SP2 [5′-GTGATCCTAACTGACCTA AGACAGG-3′]) were used successively in combination with 2 Semi-Arbitrary Degenerate primers (LAD1-1 and LAD1-3) for the initial PCR reaction, and a 16-mer primer (AC1) for the secondary reaction. The design of primer LAD1-1, LAD1-3 and AC1, the specific experimental operation and the thermal conditions for hiTAIL-PCR were all described in previous report^24^.

### The liquid enrichment approach and flow cytometric analysis

The transformation of helper and donor plasmids followed the two-component assay. After cell recovery, 4 mL cell culture (combining the two electroporation recovery cultures) was inoculated into 200 mL YND medium, resulting in an initial OD_600_ around 0.1. After 80–100 hours cultivation, the final OD_600_ would usually reach 7–9. After that, 5 μL cell culture was plated on a 15-cm YND plate lacking histidine and incubated for 48 hours. For each *TcB* pool, 20–24 separate electroporation were conducted and 10–12 plates were collected after liquid enrichment. For each *SB* pool, 50–80 separate electroporation were conducted. Since the transposition efficiency of *SB* transposon was relatively lower, 5 liquid enrichment cultures (containing 10 original electroporation) were mixed thoroughly and 5 μL cells were plated. As a result, 5–8 plates were collected for each *SB* pool. For flow cytometric analysis, GS115 and GS115-*GFP-HIS4* cells were mixed with different ratio and inoculated into YND with initial OD600 = 0.1. After plating, colonies were washed and collected from plates and fow cytometric analysis was performed on a FACSCalibur system (Becton Dickinson, Franklin Lakes, NJ), equipped with a 630 nm diode laser and a 488 nm argon laser. The specific experimental operation was described in previous report^48^.

### Transposon library generation for Illumina sequencing

To prepare DNA from the transposon library for Tn-seq analysis, cells from each mutant pool were washed from plates and around 1 × 10^8^ cells were used for genomic DNA extraction using the DNeasy Blood & Tissue Kit (Qiagen); 50~100 µL 50 µg/100 µL genomic DNA was randomly fragmented to 200–500 bp pieces by sonication with a Bioruptor® Plus which will be suitable for downstream ligations and PCR. After sonication, the genomic fragments were end repaired using the NEBNext End Repair module (New England Biolabs) in order to effectively ligate on adaptors for amplification and sequencing, and A-tails were added by incubation with Taq polymerase with 0.2 mM dATP at 72 °C for 30 minutes to allow the ligation of T-tailed adapters. To generate T-tailed adapter, Index fork R (5′-GTGACTGGAGTTCAGACGTGTGCTCTTCCGATCTGGTCGTGGTA T-3′) and 5PforktruncatedNH2 (5′-p-TACCACGACCAGATCGGA-NH2-3′) were pre-annealed by heating to 94 °C for 2 minutes in the presence of Clonetech PCR Buffer (Clonetech), cooling to 80 °C for 10 minutes, and continuously decreasing by 10 °C every 10 minutes until 20 °C. T-tailed adapter was ligated to the A-tailed fragments with T4 DNA ligase (New England Biolabs) overnight at 16 °C. The adapter-ligated fragments were PCR-amplified to enrich transposon-chromosome junctions using primer pair P7-Barcode-index-R (5′-CAAGCAGAAGACGGCATACGAGATNNN NNNGTGACTGGAGTTCAGACGTGTGC-3′)/P5-TcBR1 (5′-AATGATACGGCGA CCACCGAGATCTACACTCTTTCCCTACACGACGCTCTTCCGATCTAAATATCTCGACAAAGGGTTCCG-3′) or P5-SBR1 (5′-AATGATACGGCGACCACCGAGA TCTACACTCTTTCCCTACACGACGCTCTTCCGATCTAAATGTATTTGGCTAAGGTGTATG-3′). Amplification was performed with the following parameters: 98 °C for 30 s; 22 cycles of 10 s at 98 °C, 20 s at 60 °C and 30 s at 72 °C; 72 °C for 8 min; hold for 4 °C. To minimize bias introduced by PCR, 400 µL PCR mixture was divided into 20 aliquot parts. The PCR products of 250-450 bp were gel-purified using the Qiagen Gel extraction kit (Qiagen) and sequenced on the Illumina HiSeq X Ten platforms. (Bioproject_accession Number at NCBI: PRJNA414726, Biosample_accession Number at YND condition: SAMN07822238, Biosample_accession Number at YNM condition: SAMN07822259).

### Analysis and mapping of deep sequencing data

Deep sequencing reads from transposon-chromosome junctions of various transposon insertion libraries were trimmed to remove 5′ transposon sequence and 3′ adapter sequence using skewer (v0.2.2)^31^. Take a *TcB* insertion library as an example, if transposon-chromosome junctions were captured using primer P5-TcBR1 containing AAATATCTCGACAAAGGGTTCCG, reads containing a 5′ end sequence matching the terminus of TcBuster AATATCTCGACAAAGG were trimmed to remove the prefix, and those reads without transposon sequence were stripped off completely (command line options: skewer -r 0.05 -m head -x AAAGGTTGAAGAACACTG -L 140 raw_data_file -o trim_file1); subsequently, we used the terminal sequence of *TcB* to further trim the production by the command “skewer -r 0.05 -m head -x AAAGGTTGAAGAACACTG -L 110 trim_file1 -o trim_file2”, which excluded the sequences produced by nonspecific amplification; at last, we further trimmed the remaining reads to remove adapter sequence ATACCACGAC from the 3′ end (skewer -Q 40 -m tail -x ATACCACGAC -l 16 -L 50 -e trim_file2 -o trim_file3). The final sequence was a short fragment sequence from genomic DNA, which flanked the transposon sequence. We mapped those fragment sequences to the *Komagataella phaffii* GS115 genomic sequence (GenBank Unit Accession GCA_000027005.1) to identify insertion sites using bowtie alignment software^49^ with command line options settings: bowtie -v 3 -a–best–strata -m 1. We abandoned the reads which were mapped to more than one site. The poor-quality sequencing reads were further removed and then read counts in each insertion location were calculated using python script.

### Classification of gene essentiality

Sequence reads of each transposon insertion locus fluctuated very significantly, for instance, many loci had one read, whereas some loci had more than ten thousands reads. Although the read counts may represent the proliferation rates of the cells to some extent, they may also be strongly influenced by locus bias of our transposon system, PCR amplification bias of transposon-genome junctions or artifacts of the sequencing process. In order to use transposon insertion to analyze gene essentiality more accurately, we developed the GII, which does not take account of read counts as a main factor to classify essential genes.

1 $${\rm{GII}}=\frac{{\rm{No}}.\,\mathrm{of}\,\mathrm{Insertions}\,\ast \,72}{{\rm{No}}.\,\,\mathrm{of}\,\mathrm{TA}\,\mathrm{sites}},$$

72 is the average number of TA sites in each gene.

Nevertheless, we still utilized the read density, which is generated through dividing the number of total reads in each gene by its TA counts, as a secondary factor since it may have certain relationship with gene’s fitness. It should be stressed that, insertions in the last 10% of the gene length (at the 3′ end) were excluded when we calculated the insertion number of one gene, because it is possible that insertions close to the end of a gene had little effect on functionality^27^. We combined these sources of information (GIIs and read density) with machine learning algorithm logistic regression approach: we assigned the GII and read density of the 150 gene samples to the feature matrix X (input signal in training) and the corresponding class labels of the gene essentiality to the vector y (output signal). With an appropriate learning rate and regularization parameter (C = 10), we used Logistic Regression module from the Scikit-learn python package^50^ to fit the model on the training data which had been standardized. Consequently, we got a logistic equation which could calculate the probability of non-essential event:

2 $${\rm{y}}=\frac{1}{1+{e}^{-(5.737{x}_{1}+0.082{x}_{2}+2.098)}}$$

where ${x}_{1}$ is the standardized value of GII, ${x}_{2}$ is the standardized value of read frequency (read density), and y means the probability of particular sample belonging to non-essential gene (class 1). The y value is then considered as NEP in the results section. Thus far, we successfully worked out the NEP which can reflect gene essentiality of each gene using the above logistic regression equation.

### Spotting assay of yeast growth and colorimetrical assay of Aox

The strains were pre-grown in YPD media to OD_600_ of 4–10. The cells were harvested by centrifugation at 3,000 *g* for 5 min, washed three times with sterile water. For spotting assay of yeast growth, the washed cell pellets were then diluted to OD_600_ of 0.01, 0.1, and 8 μl of each was spotted onto solid culture plates containing indicated media. For colorable reaction of Aox, the washed cell pellets were re-suspended with initial OD_600_ of 1.0 in 50 mL YNMH media. At suitable intervals, OD_600_ was measured for growth curve, 1 mL aliquot of culture media was removed, and cells were harvested by centrifugation and then stored at −80 °C for colorimetrical assay of Aox activities. The preparation of reaction buffers and manipulation protocols were described previously^51^.

## Electronic supplementary material

Figure S1-S6

Table S1-S6

Dataset S1

Dataset S2

Dataset S3

Dataset S4

Dataset S5

Dataset S6
