# Supplementary material for: Genome-Wide Determination of Gene Essentiality by Transposon Insertion Sequencing in Yeast Pichia pastoris
Source: Sci Rep. 2018 Jul 5;8:10223. doi: 10.1038/s41598-018-28217-z (PMC6033949; doi:10.1038/s41598-018-28217-z)
Supplement: Supplementary file 1 — Figure S1-S6 [file 41598_2018_28217_MOESM1_ESM.pdf]

Genome-Wide Determination of Gene Essentiality by Transposon Insertion Sequencing in Yeast *Pichia pastoris*

Jinxiang Zhu[1], Ruiqing Gong[1], Qiaoyun Zhu[1], Qiulin He[1], Ning Xu[1], Yichun Xu[1], Menghao Cai[1], Xiangshan Zhou[1], Yuanxing Zhang[1,2] and Mian Zhou[1,*]

[1] State Key Laboratory of Bioreactor Engineering, East China University of Science and Technology, Shanghai 200237, China

[2] Shanghai Collaborative Innovation Center for Biomanufacturing (SCICB), Shanghai 200237, China

**Corresponding author: E-mail for Mian Zhou: mianzhou@ecust.edu.cn*

Figure S1

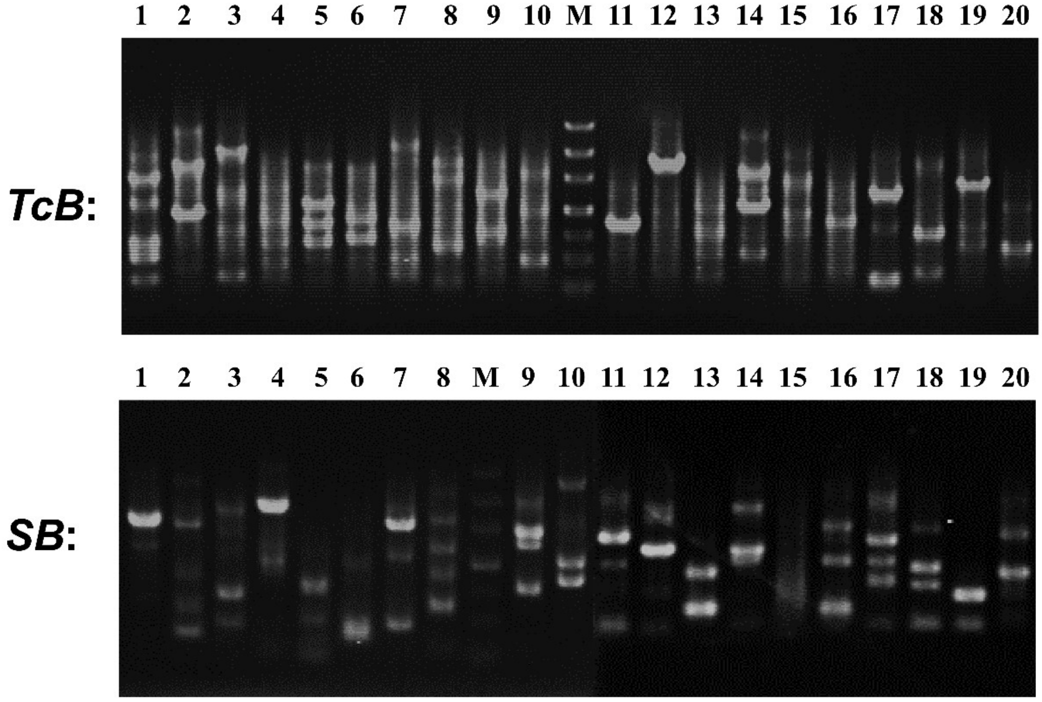

Figure S1. High-efficiency thermal asymmetric interlaced PCR (hiTAIL-PCR) analysis of His+ transformants. (**A**) Amplification of TcBTIRsHis flanking sequences from host genome by hiTAIL-PCR. (**B**) Amplification of SBTIRsHis flanking sequences from host genome by hiTAIL-PCR.

Figure S2

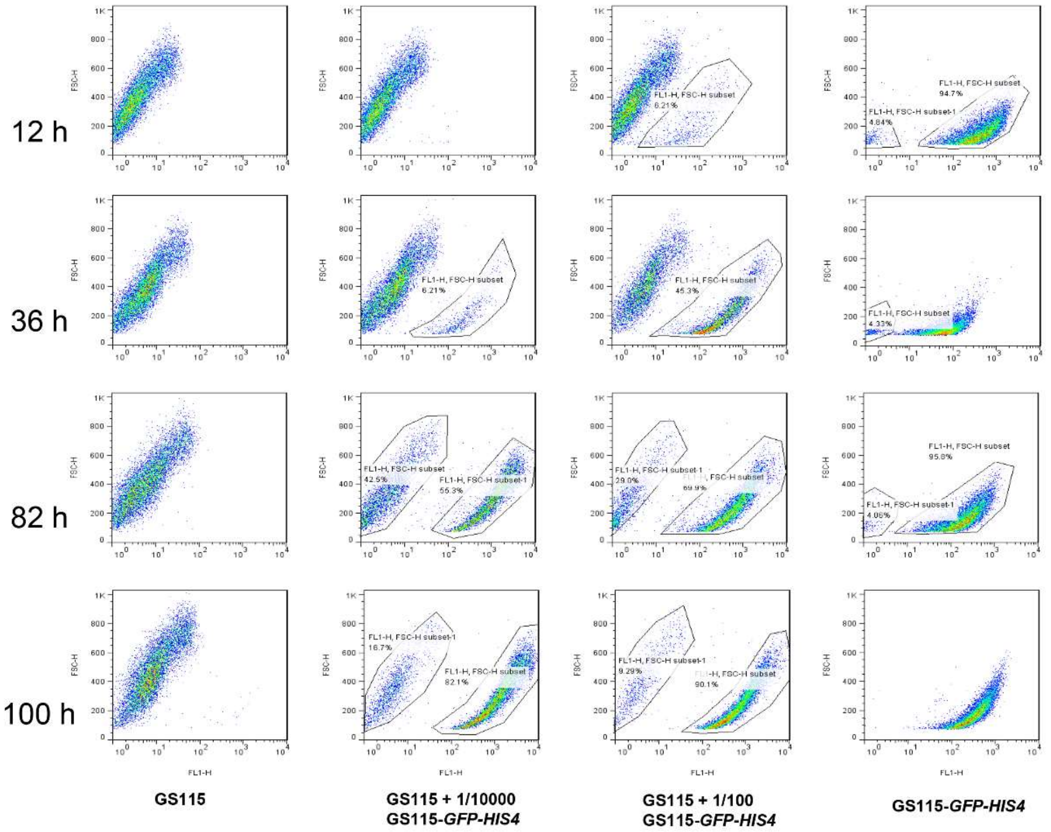

Figure S2. Flow cytometric analysis of the ratio of GS115 and GS115-*GFP-HIS4* cells at different time points.

Figure S3

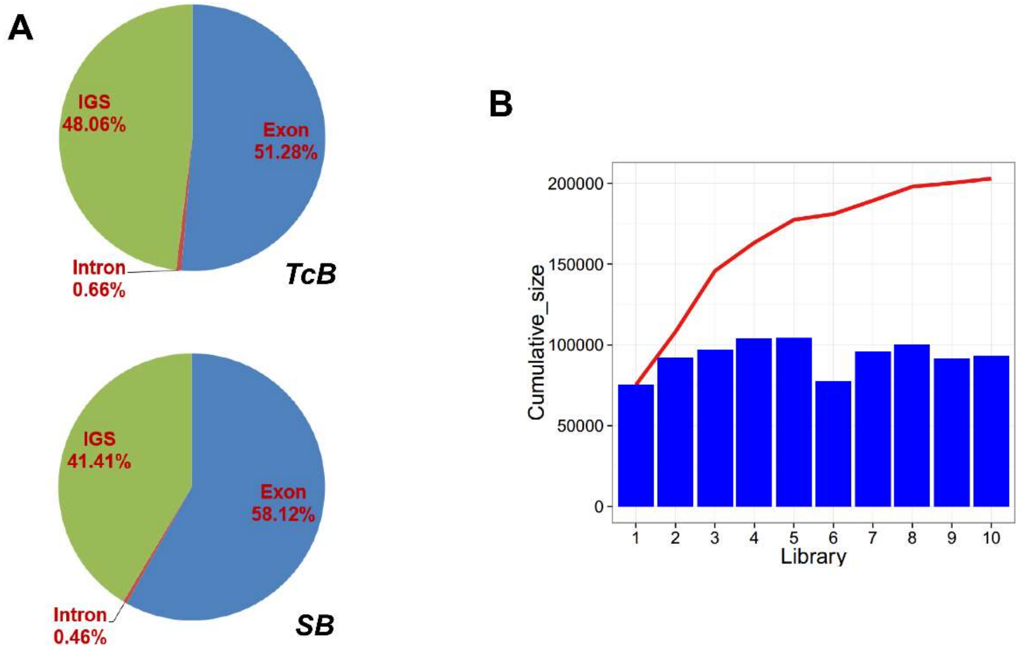

Figure S3. (**A**) Distributions of *TcB* (top panel) and *SB* (bottom panel) insertions in intergenic regions, exons and introns of *P. pastoris*. (B) Saturation analysis of our Tn-seq data sets. The blue bars show the saturation level of the individual subsets. Cumulative saturation levels of insertions are represented by the red line.

Figure S4

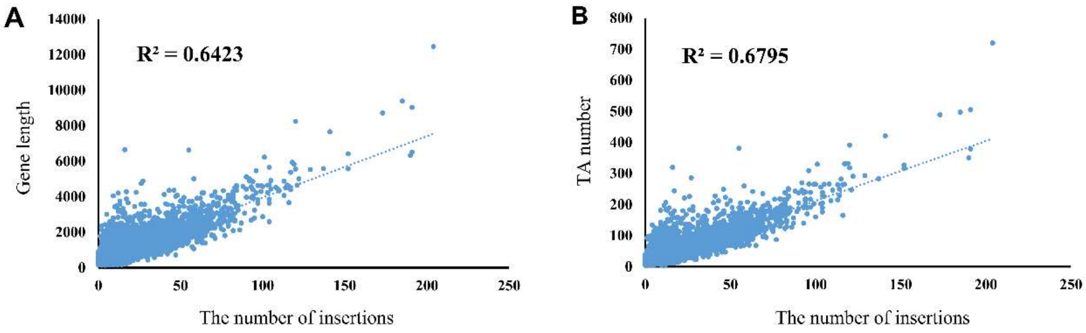

Figure S4. (**A**) The overall correlation between insertions per gene and gene length. (B) The overall correlation between insertions per gene and TA number.

Figure S5

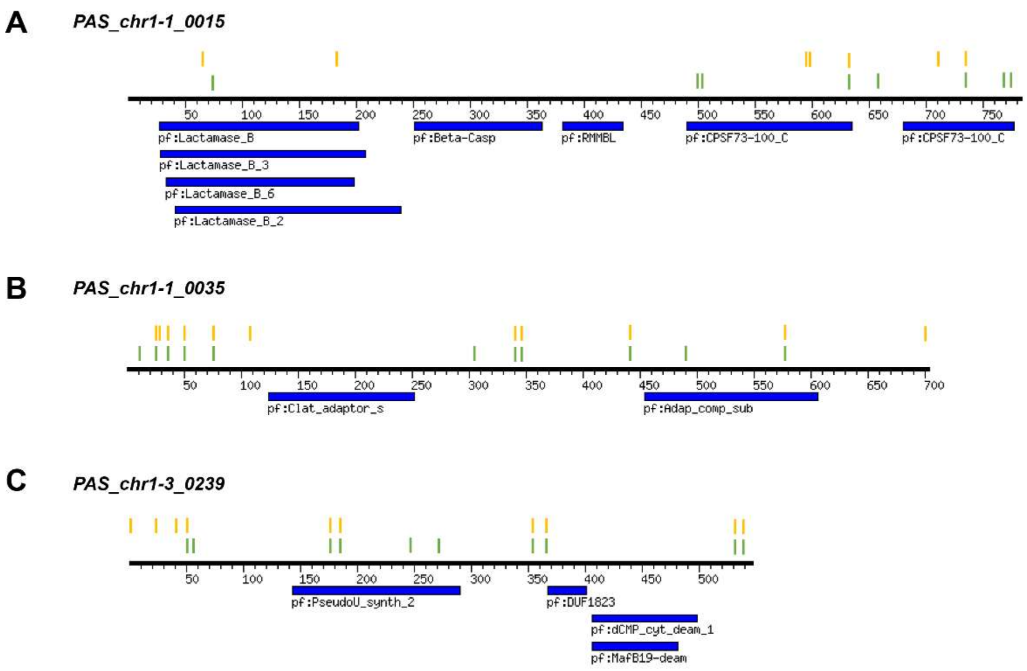

Figure S5. The transposon insertion map and domain positions of *PAS_chr1-1_0015* (A), *PAS_chr1-1_0035* (B) and *PAS_chr1-3_0239* (C). Motifs were predicted by SSDB motif search tool on KEGG website.

Figure S6

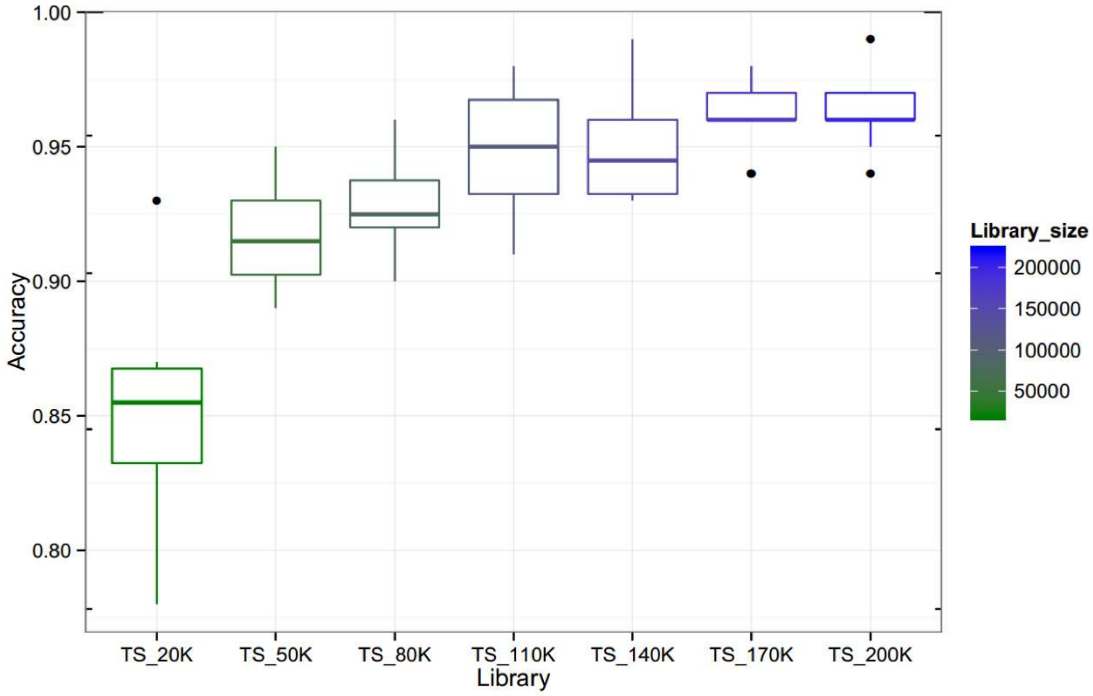

Figure S6. The training accuracy of insertion subsets with different saturation.
