# Supplementary material for: Genome-Wide Determination of Gene Essentiality by Transposon Insertion Sequencing in Yeast Pichia pastoris
Source: Sci Rep. 2018 Jul 5;8:10223. doi: 10.1038/s41598-018-28217-z (PMC6033949; doi:10.1038/s41598-018-28217-z)
Supplement: Supplementary file 2 — Table S1-S6 [file 41598_2018_28217_MOESM2_ESM.pdf]

Genome-Wide Determination of Gene Essentiality by Transposon Insertion Sequencing in Yeast *Pichia pastoris*

Jinxiang Zhu[1], Ruiqing Gong[1], Qiaoyun Zhu[1], Qiulin He[1], Ning Xu[1], Yichun Xu[1], Menghao Cai[1], Xiangshan Zhou[1], Yuanxing Zhang[1,2] and Mian Zhou[1,*]

[1] State Key Laboratory of Bioreactor Engineering, East China University of Science and Technology, Shanghai 200237, China

[2] Shanghai Collaborative Innovation Center for Biomanufacturing (SCICB), Shanghai 200237, China

**Corresponding author: E-mail for Mian Zhou: mianzhou@ecust.edu.cn*

Table S1. Primers and PCR templates for plasmid construction

Primer name	Primer sequences (5'-3')	PCR template	Amplification products	Logogram
THI11p2-amp-F	AATAACAGTTATTATTCGCAAGTTCCGGTACAAGCGTGC	GS115 genome DNA	PpTHI11p fragment	Promoter fragment
THI11p-R	CATGATGATTTATTGAAGTTTCC			
PpMET3p-amp-F	AATAACAGTTATTATTCGTTCAGGCAACAGGACCCTCC	GS115 genome DNA	PpMET3p fragment	
PpMET3p-R	TTCTTTCTGAGTTGGTTTC			
PpAOX1p-amp-F	AATAACAGTTATTATTCGCGAGATCTAACATCCAAAGAC	GS115 genome DNA	PpAOX1p fragment	
PpAOX1p-R	CGTTTCGAATAATTAGTTGT			
pG-A1-F	TAAGTTTTAGCCTTAGACATGAC	pPICZalpha B*	AOX1TT fragment	
AOX1TT-A1-R	GGATCCGCACAAACGAAG			
His-starg-F	ATTCTGCGAGAAGGTCCTGGATGCTGTAGGCATAG	pPIC3.5K*	His4 fragment	
His-starg-R	CTTCGTTTGTGCGGATCACACTGGCAGAGCATTACGC			
Himarase-met3p-F	CCAACTCAGAAAGAAATGGAAAAAAAGGAATTTCGTG	pMAR 2XT7[1]	Himarase fragment	Transposase fragment
Himarase-aox1tt-R	TCTAAGGCTAAAACTTATTCAACATAGTTCCCTTCAAG			
SBase-met3p-F	ACCAACTCAGAAAGAAATGGGAAAATCAAAAGAAATC	pCMV(CAT)T7-SB100[2]	SBase fragment	
SBase-aox1tt-R	TCTAAGGCTAAAACTTAGTATTTGGTAGCATTGC			
Osmarase-met3p-F	ACCAACTCAGAAAGAAATGCAAGAGTACGGCGTGT	pOsm14Tpase[3]	Osmarase fragment	
Osmarase-aox1tt-R	TCTAAGGCTAAAACTTACTGCACTTGGTTGGCTAA			
TcBase-met3p-F	ACCAACTCAGAAAGAAATGATGCTGAATTGGCTGAAA	pGALS-TcBusterCOHyper[4]	TcBase fragment	
TcBase-aox1tt-R	TCTAAGGCTAAAACTTAGTGAGATTTCTGGGCCTGC			
Mosase-met3p-F	ACCAACTCAGAAAGAAATGTCGAGTTTCGTGCCG	pCFJ601 - Peft-3 Mos1 transposase[5]	Mosase fragment	
Mosase-aox1tt-R	TCTAAGGCTAAAACTTATTCAAAGTATTTGCCGTCG			
HimarL-amp-F	ATAACAGTTATTATTCGTAACAGGTTGGCTGATAAGTC	pMAR 2XT7	HimarTIRL fragment	TIRL fragment
HimarL-arg4-R	GGACCTTCTCGCAGAATCAAGCAGATTACGGTGACG			
SBL-amp-F	ATAACAGTTATTATTCGAATTGGAGCTCGGATCCCTA	pT2-shp53/GFP4[6]	SBTIRL fragment	
SBL-arg4-R	GGACCTTCTCGCAGAATCTATGGCTCGTACTCTATAG			
OsmarL-amp-F	ATAACAGTTATTATTCGTGAGGTCTGTTTACTCCCTCCG	pT7-GFP-oen[7]	OsmarTIRL fragment	
OsmarL-arg4-R	GGACCTTCTCGCAGAATGTAAAACGAGAAATTTTATTCC			
TcBL-amp-F	ATAACAGTTATTATTCGCTTTAGGCCAGTGTTCTTCAAC	pRS416 URA3 mini TcBuster-ClonNAT[4]	TcBTIRL fragment	
TcBL-arg4-R	GGACCTTCTCGCAGAATATTTCTGAACGATTCTAGGTT			
MosL-amp-F	ATAACAGTTATTATTCGTTTTGGTCATGCATGAGATC	pJL43.1 - Pglh-2::MosTase::glh-2utr[5]	MosTIRL fragment	
MosL-arg4-R	GGACCTTCTCGCAGAATTAGCTTGGCGTAATCATGG			
HimarR-arg4-F	GATCCGCACAAACGAAGACCGAGATAGGGTTGAGTG	pMAR 2XT7	HimarTIRR fragment	TIRR fragment
HimarR-pbr322-R	CTGGCCTTTTGCTCACATAACAGGTTGGCTGATAAGTC			
SBR-arg4-F	GATCCGCACAAACGAAGAGATCTAGCTTGTGGAAGGC	pT2-shp53/GFP4	SBTIRR fragment	
SBR-pbr322-R	CTGGCCTTTTGCTCACAGACTCTAGCTAGAGGATCCC			
OsmarR-arg4-F	GATCCGCACAAACGAAGGTGCTGAGGGTAGGTAGGAT	pT7-GFP-oen	OsmarTIRR fragment	
OsmarR-pbr322-R	CTGGCCTTTTGCTCACAAACACTAAACCGTTTACTCC			
TcBR-arg4-F	GATCCGCACAAACGAAGTTTTTATTTTTTATTTTATATATTAT	pRS416 URA3 mini TcBuster-ClonNAT	TcBTIRR fragment	
TcBR-pbr322-R	CTGGCCTTTTGCTCACAGTATAAAGCAGTGTTCTTCAACC			

MosR-arg4-F	GATCCGCACAAACGAAGTGCCGGATCTGCGGCTTA	pJL43.1 - Pglh-2::MosTase::glh-2utr	MosTIRR fragment
MosR-pbr322-R	CTGGCCTTTTGCTCACAGGATCCAACAAAAAAAGCACT		
pG-A1-F	TAAGTTTTAGCCTTAGACATGAC	pPICZalpha B	AOX1TT-Zeocin-pBR322 fragment
pBR-ampr2-R	CGAATAATAACTGTTATTCATGACCAAAATCCCTTAACG		
pG-A2-F	TGTGAGCAAAAGGCCAGC	pPIC3.5K	pBR322-Amp
Amp-L1-R	CGAATAATAACTGTTATTTTTCAGTG		
pAG32-A1-F	CAAGGGTCTCGAGATAAGAAGCCAGGTTAACCTGCATTAATG	pAG32[7]	pAG32Linear fragment
pAG32-A1-R	CAAATCAAAATCAAACTGATCGAAATAGCTCGTTTTCGACAC		
DAS1TT-R	CTTATCTCGAGACCCTTGTGACTGACACTT	pPpT4_pHTX1-HsCas9-GUT1-gRNA2[8]	HXT1-HsCas9 fragment
HTX1-R	TCAGTTTGATTTTGATTTGTTTAGGTAACT		
Zeocin-pars-F	GTTGAGTCGACATCGAGGATCCCCCACACACCATAG	pPICZalpha B	pDZ-Zeocin fragment
pDZ-htx1-R	CACAAGGGTCTCGAGATAGATCTCATGCATGACCAAAATC		
PARS-F	CTCGAACATAGTCCGTCCC	GS115 genome DNA	PARS fragment
PARS-R	TCGATGTCGACTCAACCTAT		
HTX1-F	TCGAGACCCTTGTGTGTTGTAGTTTTAATATAGTT	pPpT4_pHTX1-HsCas9-GUT1-gRNA2[8]	HXT1-gRNA-AOX1TT fragment
AOX1TT-pars-R	ACGGACTATGTTCGAGGATCCGCACAAACGAACGTCTCAC		

* Invitrogen

[1] Liberati NT1, et al. (2006) An ordered, nonredundant library of *Pseudomonas aeruginosa* strain PA14 transposon insertion mutants. *Proc Natl Acad Sci U S A* 103(8):2833-8.

[2] Mátés L, et al. (2009) Molecular evolution of a novel hyperactive *Sleeping Beauty* transposase enables robust stable gene transfer in vertebrates. *Nat Genet* 41(6):753-61.

[3] Fattash I, et al. (2013) A rice *Stowaway* MITE for gene transfer in yeast. *PLoS One* 2013, 21: 8(5)

[4] Li X, et al. (2013) A resurrected mammalian *hAT* transposable element and a closely related insect element are highly active in human cell culture. *Proc Natl Acad Sci U S A* 110(6):E478-87.

[5] Frøkjær-Jensen C, et al. (2012) Improved *Mos1* -mediated transgenesis in *C. elegans* . *Nat Methods* 9(2):117-8.

[6] Wiesner SM, et al. (2009) De novo induction of genetically engineered brain tumors in mice using plasmid DNA. *Cancer Res* 69(2):431-9.

[7] Goldstein, A.L, et al. (1999) Three new dominant drug resistance cassettes for gene disruption in *Saccharomyces cerevisiae* . *Yeast* 15, 1541-15.

[8] Weninger A, et al. (2016) Combinatorial optimization of CRISPR/Cas9 expression enables precision genome engineering in the methylotrophic yeast *Pichia pastoris* . *J Biotechnol* 235:139-4

Table S2. ***TcB* and *SB* insertion sites in *P. pastoris***

Strains	Chromosome	Chromosome position	Target site duplication	Insertion direct	Insertion gene
TcB					
1	Chr. I	276208	CCA**TA**GTA	-	PAS_chr1-3_0149
2	Chr. II	1325749	ATT**TA**GAG	-	
3	Chr. IV	444217	ATC**TG**GGA	+	
4	Chr. I	2305281	GTT**TA**TGT	-	PAS_chr1-4_0495
5	Chr. III	1510286	ACC**TA**TGC	+	PAS_chr3_0787
6	Chr. III	852296	CAC**TA**GAG	-	
7	Chr. II	2004675	CTA**TA**GAT	+	
8	Chr. II	1393275	GGC**TA**AAC	-	PAS_chr2-1_0738
9	Chr. I	1297232	AGC**TA**GAC	-	
10	Chr. II	1260528	TTT**TA**TGT	+	
11	Chr. III	1470103	CTC**TA**AAA	-	
12	Chr. I	2781575	CTC**TA**GAC	+	
13	Chr. I	94889	GTT**TA**AAA	-	
14	Chr. III	956286	GTC**TA**AAG	+	PAS_chr3_0501
15	Chr. I	1193817	CTA**TA**AGC	-	
16	Chr. IV	116970	CCG**TA**AAG	-	
17	Chr. III	654386	GTT**TA**ACA	-	PAS_chr3_0332
18	Chr. II	1313009	ATC**TA**GTT	-	PAS_chr2-1_0878
19	Chr. IV	962371	CTT**TA**ATC	+	PAS_chr4_0489
20	Chr. II	1906198	CTC**TA**TGC	+	PAS_chr2-2_0260
SB					
1	Chr. II	2164550	TA	+	PAS_chr2-2_0115
2	Chr. I	2162578	TA	+	
3	Chr. II	1892497	TA	-	PAS_chr2-2_0267
4	Chr. III	1517716	TA	-	PAS_chr3_0792
5	Chr. II	325387	TA	+	PAS_chr2-1_0174
6	Chr. III	1071594	TA	+	PAS_chr3_0561
7	Chr. II	420743	TA	-	
8	Chr. II	589051	TA	-	
9	Chr. IV	80048	TA	+	
10	Chr. II	55425	TA	+	PAS_chr2-1_0029
11	Chr. III	293469	TA	+	
12	Chr. II	370811	TA	-	
13	Chr. IV	290047	TA	+	
14	Chr. I	2212699	TA	-	PAS_chr1-4_0437
15	Chr. I	1600842	TA	+	PAS_chr1-4_0105
16	Chr. III	1523151	TA	-	PAS_chr3_0795
17	Chr. I	2745884	TA	-	
18	Chr. I	1932919	TA	+	PAS_chr1-4_0666
19	Chr. III	239054	TA	+	PAS_chr3_0117
20	Chr. I	219391	TA	+	PAS_chr1-3_0114

Table S3. Number of mutants, sequence reads and unique insertion sites for each library

Mutant pool	Mutants	Mapped reads	Unique insertion sites
TcB1	~280,000	12,480,716	66,870
TcB2	~280,000	13,875,986	64,473
TcB3	~280,000	12,911,125	62,541
TcB4	~280,000	13,025,039	63,500
TcB5	~300,000	13,303,247	68,461
TcB6	~300,000	11,433,330	69,722
TcB7	~320,000	11,710,978	71,652
TcB8	~320,000	12,886,935	76,332
TcB9	~320,000	11,733,232	72,425
TcB10	~320,000	13,252,105	78,110
SB1	~100,000	19,536,032	21,061
SB2	~100,000	5,717,466	19,544
SB3	~160,000	3,410,761	35,053
SB4	~160,000	7,076,272	34,462
SB5	~160,000	5,678,536	32,931
SB6	~160,000	5,078,631	35,551
SB7	~160,000	5,605,812	37,307
SB8	~160,000	7,618,087	33,556
SB9	~160,000	7,081,782	31,917
SB10	~160,000	8,582,528	35,714
Total	~4,480,000	201,998,600	202,858

Table S4. The proportions of the four nucleotides at target sites.

Site	Nucleotide	Proportion			Site	Nucleotide	Proportion			Site	Nucleotide	Proportion		
		TcB	SB	TcB and SB			TcB	SB	TcB and SB			TcB	SB	TcB and SB
1	A	0.21	0.29	0.25	7	A	0.21	0.21	0.22	13	A	0.21	0.22	0.22
1	T	0.33	0.31	0.32	7	T	0.43	0.36	0.39	13	T	0.25	0.64	0.44
1	G	0.21	0.21	0.21	7	G	0.13	0.21	0.18	13	G	0.3	0.05	0.18
1	C	0.25	0.19	0.22	7	C	0.22	0.21	0.22	13	C	0.24	0.09	0.17
2	A	0.31	0.29	0.3	8	A	0.19	0.44	0.31	14	A	0.26	0.27	0.27
2	T	0.24	0.31	0.28	8	T	0.3	0.18	0.24	14	T	0.25	0.29	0.27
2	G	0.26	0.21	0.23	8	G	0.13	0.21	0.17	14	G	0.26	0.2	0.23
2	C	0.19	0.2	0.19	8	C	0.38	0.17	0.28	14	C	0.23	0.24	0.24
3	A	0.48	0.29	0.38	9	A	0.02	0.01	0.01	15	A	0.59	0.26	0.42
3	T	0.37	0.3	0.33	9	T	**0.94**	**0.97**	**0.95**	15	T	0.19	0.31	0.25
3	G	0.1	0.21	0.16	9	G	0.01	0.01	0.01	15	G	0.15	0.19	0.17
3	C	0.05	0.21	0.13	9	C	0.04	0.01	0.03	15	C	0.07	0.24	0.16
4	A	0.19	0.31	0.25	10	A	**0.94**	**0.97**	**0.95**	16	A	0.37	0.3	0.33
4	T	0.6	0.26	0.42	10	T	0.02	0.01	0.02	16	T	0.48	0.29	0.38
4	G	0.07	0.24	0.16	10	G	0.04	0.01	0.02	16	G	0.05	0.2	0.13
4	C	0.15	0.19	0.17	10	C	0.01	0.01	0.01	16	C	0.1	0.21	0.16
5	A	0.25	0.29	0.27	11	A	0.3	0.18	0.24	17	A	0.24	0.3	0.27
5	T	0.26	0.26	0.26	11	T	0.18	0.44	0.31	17	T	0.31	0.29	0.3
5	G	0.23	0.24	0.24	11	G	0.39	0.17	0.28	17	G	0.19	0.2	0.2
5	C	0.26	0.2	0.23	11	C	0.13	0.2	0.17	17	C	0.26	0.21	0.23
6	A	0.25	0.63	0.43	12	A	0.43	0.35	0.39	18	A	0.33	0.31	0.32
6	T	0.21	0.22	0.22	12	T	0.21	0.21	0.22	18	T	0.2	0.29	0.25
6	G	0.24	0.09	0.17	12	G	0.22	0.22	0.22	18	G	0.26	0.19	0.22
6	C	0.3	0.06	0.18	12	C	0.13	0.22	0.18	18	C	0.21	0.21	0.21

Table S5. Mutations in genes disrupted by CRISPR/Cas9 system

Locus tag	P-value	Category	Target site	Mutation
**Essential genes validation**				
PAS_chr2-1_0631	0.025990991	Essential	AAAGTATCCTGTGTTGACCC	TTG-, GTT-, TTG-
PAS_chr3_1050	0.026048858	Essential	ATGTGACACTCCATCAAACG	CAT-, ATC-, AAA-
PAS_chr2-1_0167	0.026341799	Essential	ATGCTTCGACGACTCAGATG	GACTCA-, CTC-, CAG-
PAS_chr2-1_0456	0.026353444	Essential	CCCGTCTCTTCAGCACTACT	ACT-, CAC-, CAGCAC-
PAS_chr3_0878	0.026614121	Essential	CAACATGAGCAGAAATAACC	TAA-, ATA-, ATA-
PAS_chr1-1_0191	0.026701837	Essential	AAGCCACCAGTTCAATTGTT	TTG-, GTT-, ATT-
PAS_chr3_0997	0.026911281	Essential	TGCTTCATATGTCCTTTACC	AGA-, AGAAAT-,AAA-
PAS_chr2-1_0366	0.027065392	Essential	AGTCCGAAATTGATGACTGG	GAT-, GAC-, GAT-
PAS_chr2-2_0235	0.02719965	Essential	GTTGACCACAGCTAGAATCA	GAA-, AGA-, AGA-
PAS_chr1-4_0209	0.027343841	Essential	GCCGATATTCCATACAAGAT	CAT-, ACA-, CAT-
PAS_chr2-1_0349	0.027536422	Essential	GGATACCACACCTTAAACAA	AAC-, TTA-, TTA-
PAS_chr1-4_0063	0.027565798	Essential	TGAGAGGTACCTTGGAAGGT	AAG-, TGGAAG-, GAA-
PAS_chr4_0863	0.028177922	Essential	TGGGACTGTGTGGTTGCACA	TGT-, TTG-, TTG-
PAS_chr1-3_0108	0.028277332	Essential	AATCAGCAGGAGAGAACGTT	GAG-, AAC-, GAACGT-
PAS_chr1-3_0186	0.028889788	Essential	ATGACCTCAACACATACATG	A+, AC-
PAS_chr3_0515	0.028939966	Essential	GACCAACCCTAAAACGATAC	ACG-, CCT-, ACG-
PAS_chr4_0865	0.029052361	Essential	GGAAATACCTCAAGTGCAGT	AGT-, GCA-, GCA-
PAS_chr2-1_0160	0.029233804	Essential	GTCTTAGGTTTCCCACGTAT	C+, ACGT-
PAS_chr4_0956	0.02934039	Essential	GGTATGGTGCCAACATGAAG	TATGGTGCCAACATG-,AAA-,CATGAA-
**Non-essential genes validation**				
PAS_chr2-2_0180	0.915960201	Non-essential	TACTTGCTGAACAAATCGGG	G+
PAS_chr3_1036	0.920069135	Non-essential	AGGCAACATTAGTGCCGATC	CG-
PAS_chr1-4_0272	0.957357059	Non-essential	GCTTCGTGCTTCAGTGTGCA	GT-
PAS_chr4_0212	0.958519434	Non-essential	AGAATTATAGGAGTTGGGTC	G-
PAS_chr3_0584	0.96877591	Non-essential	AATTACCGCCTTAGCCGCAT	C-
PAS_chr1-1_0055	0.977529915	Non-essential	GTACTCTATTTATGAGCACC	GC-
PAS_chr1-1_0319	0.979367754	Non-essential	TGTAACGTCCTCTTCTACAG	T-
PAS_chr1-3_0142	0.981789539	Non-essential	CGCTAAAGGTGAACCGATTC	A+
PAS_chr1-4_0228	0.982379027	Non-essential	TCTTGTTAACCGTCCAAAAG	A-
PAS_chr3_0423	0.985371015	Non-essential	TCATGTTGCTACTGCTCGCG	A+
PAS_chr1-3_0113	0.998142015	Non-essential	GTTGTAGGTGACGGTGCTGT	C-
PAS_chr4_0880	0.998486286	Non-essential	TTTGATCCGTTCGCCGATAC	G-
PAS_chr2-2_0005	0.998842022	Non-essential	ATTCATCACACTTCATCGCA	A+
PAS_chr4_0881	0.999955709	Non-essential	GCTTCTGAAGAACCAAGAAA	A-
PAS_chr2-2_0365	0.999997504	Non-essential	CTCCGAAGATATAGAGGGTC	G-
PAS_chr2-2_0435	0.999997567	Non-essential	AGAGCAGATAGAGAAACTGC	T-
PAS_chr4_0259	0.999999663	Non-essential	TCCTACTGCAAAGTACAGCG	AGCG-
PAS_chr3_0693	0.999999999	Non-essential	TTCGTCACGGCCAATCTCAA	CT-

Table S6. Novel methylotrophic functional genes

Locus tag	P-value of YND	P-value of YNM	Target site	Mutation
PAS_chr3_0072	0.958218261	0.01215503	ORF	deletion
PAS_chr1-4_0498	0.980592211	0.237770405	ORF	deletion
PAS_chr1-3_0278	0.982058797	0.01215503	CACTGGAGCTTATCTCAACA	ATCTC-
PAS_chr2-1_0748	0.990982561	0.028365249	ATGAGCAAACAAACTCCGTC	TC-
PAS_chr3_0968	0.945948673	0.10067283	CAAGGCAACCCTGTCTCTGC	T+
PAS_chr3_0689	0.971505746	0.321835936	ACGAACTCTCACAGACATAA	C-
PAS_chr2-2_0202	0.948634657	0.426929721	AATGAGTAGTTGGATTCCTC	C-
PAS_chr4_0887	0.921405674	0.478682398	ATTACTACAAAAGGGGCGAA	A+
